# Iridates from the molecular side

**DOI:** 10.1038/ncomms12195

**Published:** 2016-07-20

**Authors:** Kasper S. Pedersen, Jesper Bendix, Alain Tressaud, Etienne Durand, Høgni Weihe, Zaher Salman, Thorbjørn J Morsing, Daniel N. Woodruff, Yanhua Lan, Wolfgang Wernsdorfer, Corine Mathonière, Stergios Piligkos, Sophia I. Klokishner, Serghei Ostrovsky, Katharina Ollefs, Fabrice Wilhelm, Andrei Rogalev, Rodolphe Clérac

**Affiliations:** 1 CNRS, CRPP, UPR 8641, Pessac 33600, France; 2 Univ. Bordeaux, CRPP, UPR 8641, Pessac 33600, France; 3 CNRS, ICMCB, UPR 9048, Pessac 33600, France; 4 Univ. Bordeaux, ICMCB, UPR 9048, Pessac 33600, France; 5 Department of Chemistry, University of Copenhagen, Copenhagen DK-2100, Denmark; 6 Laboratory for Muon Spin Spectroscopy, Paul Scherrer Institut, Villigen PSI CH-5232, Switzerland; 7 Department of Chemistry, University of Oxford, Oxford OX1 3QR, UK; 8 CNRS, Inst NEEL, Grenoble F-38000, France; 9 Institute of Applied Physics, Academy of Sciences of Moldova, Kishinev 2028, Moldova; 10 ESRF - The European Synchrotron, CS 40220, 38043 Grenoble Cedex 9, France

## Abstract

New exotic phenomena have recently been discovered in oxides of paramagnetic Ir^4+^ ions, widely known as ‘iridates'. Their remarkable properties originate from concerted effects of the crystal field, magnetic interactions and strong spin-orbit coupling, characteristic of 5d metal ions. Despite numerous experimental reports, the electronic structure of these materials is still challenging to elucidate, and not attainable in the isolated, but chemically inaccessible, [IrO_6_]^8–^ species (the simplest molecular analogue of the elementary {IrO_6_}^8−^ fragment present in all iridates). Here, we introduce an alternative approach to circumvent this problem by substituting the oxide ions in [IrO_6_]^8−^ by isoelectronic fluorides to form the fluorido-iridate: [IrF_6_]^2−^. This molecular species has the same electronic ground state as the {IrO_6_}^8−^ fragment, and thus emerges as an ideal model for iridates. These results may open perspectives for using fluorido-iridates as building-blocks for electronic and magnetic quantum materials synthesized by soft chemistry routes.

## Abstract (web summary)

Iridates are known to exhibit a range of exotic electronic and magnetic behaviours but it is difficult to prepare isolated [IrO_6_]^8−^ species via soft chemical routes. Here, the authors isolate the isoelectronic [IrF_6_]^2−^ complex, and assess it as a model and for iridate analogues.

## Graphical abstract

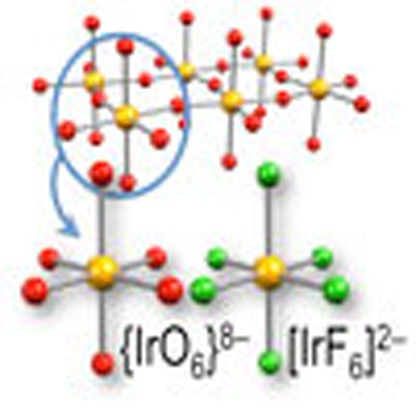

The 5d elements of the periodic table possess singular properties including a strong coupling of the electronic spin to its orbit as well as spatially extended valence orbitals leading to a reduced electronic repulsion and large effect on the ligand field. These intrinsic characteristics have been experimentally or theoretically demonstrated to be responsible for new exotic states of matter such as spin-orbit (SO) Mott insulators^1^,^2^, topological insulators^3^,^4^,^5^, super-conductors^6^,^7^,^8^, spin–liquids and –ices^9^,^10^,^11^ and for quantum metal–insulator transitions^12^. The common denominator in these promising materials, collectively referred to as ‘iridates' (herein named oxido-iridates), is the presence of octahedrally coordinated Ir^IV^ ions (Fig. 1a,b) featuring a t_2*g*_^5^ electronic configuration. The half-filled *j*_eff_=1/2 level (Fig. 1c)^13^, resulting from the SO coupling, is at the origin of a narrow band gap responsible for the abovementioned phenomena. In all oxido-iridates including Sr_2_IrO_4_, the archetypal SO Mott insulator^2^, corner- or edge-sharing {IrO_6_}^8−^ distorted octahedra are present (Fig. 1a). While magnetic Ir^IV^–Ir^IV^ interactions and their implications for the physical properties have been studied extensively in oxido-iridates^14^,^15^, the intrinsic magnetic properties^16^ of the elemental {IrO_6_}^8−^ moiety are consistently masked by the same long-range interactions. Birol and Haule^17^ recently suggested that the design of materials incorporating isolated Ir^IV^ octahedra should facilitate smaller bandwidths and promote Mott ground states. If this strategy is developed to its logical end, a discouraging result is obtained as the isolation of the [IrO_6_]^8−^ ion (Fig. 1b) is chemically impossible. However for the realization of dimensionally reduced oxido-iridates, the fluoride ion appears as an ideal substitute to oxide being isoelectronic with comparable chemical and physical characteristics, but, importantly, with a reduced charge. Along this idea, we present the synthesis of molecular fluorido-iridates incorporating spatially isolated [IrF_6_]^2−^ units. Their local magnetic properties are probed by X-ray magnetic circular dichroism (XMCD) spectroscopy demonstrating that the [IrF_6_]^2−^ and {IrO_6_}^8−^ units possess virtually identical electronic ground states as suggested by theory^17^. Thus this fluorido-iridate moiety and its intrinsic properties, experimentally determined in this work, can be confidently used to model and emulate the basic {IrO_6_}^8−^ unit in oxido-iridates.

## Results

### Syntheses and structures

Highly water-soluble Na_2_[IrF_6_] was obtained by direct F_2_ fluorination of Na_2_[IrCl_6_]·6H_2_O at elevated temperature (200 °C) and characterized by powder X-ray diffraction^18^,^19^,^20^. Addition of a solution of PPh_4_Cl (PPh_4_^+^=tetraphenylphosphonium(V) cation) to an aqueous [IrF_6_]^2−^ solution yielded (PPh_4_)_2_[IrF_6_]·2H_2_O (**1**, Fig. 2a and Supplementary Fig. 1). Electrospray mass spectrometry on **1** indicated complete absence of any hydrolysis products such as [IrF_5_(OH)]^2−^ (Supplementary Fig. 2). **1** was subsequently used to synthesize Zn(viz)_4_[IrF_6_] (**2**, Fig. 2b and Supplementary Fig. 3; viz=1-vinylimidazole). These compounds incorporate a low symmetry, but close-to-octahedral [IrF_6_]^2−^ unit in **1** (*P*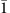 space group) and a tetragonally distorted octahedral [IrF_6_]^2−^ unit in **2** (*P*4_2_/*n* space group). The axial elongation in both **1** and **2** is ∼1%, slightly smaller than, for example, the ∼3% found in Sr_2_IrO_4_, and the bond angles are all within 1.5 and 0.1% of 90°, respectively. To elucidate any differences in the Ir^IV^ electronic structure when modifying the ligand field, we included in this study the related chloride complex, (PPh_4_)_2_[IrCl_6_] (**3**, Fig. 2c and Supplementary Fig. 4), also featuring an approximately octahedral, but axially compressed (∼0.7%) [IrCl_6_]^2−^ ion. The nearest neighbour Ir–Ir distances (Å) are 10.0 (**1**), 8.1 (**2**) and 10.1 (**3**), which are much longer than in the oxido-iridates (for example, 3.9 Å in Sr_2_IrO_4_). Thus the Ir^IV^–Ir^IV^ interactions are negligibly small and the [IrX_6_]^2−^ (X=F, Cl) unit can be considered as magnetically isolated in **1**, **2** and **3**.

### Magnetic properties and X-ray spectroscopy

The susceptibility-temperature product, *χT*, of **1**–**3** (Supplementary Fig. 5) is practically temperature independent (Curie's law) confirming the lack of significant magnetic interactions between Ir^IV^ spins and thus the absence of any magnetic order at least down to 1.8 K. The complete set of magnetization data (*M* versus *μ*_0_*HT*^–1^; Supplementary Figs 6–8) could be fitted to the Brillouin function for an effective spin-1/2 (with *g*≈2), which corroborates the Curie susceptibility and demonstrates the presence of an energetically isolated *J*_eff_=1/2 ground state.

Orbital (*M*_orbital_) and spin (*M*_spin_) contributions to the total magnetic moments (*M*_total_) have been determined experimentally using XMCD, which is defined as the difference between two X-ray absorption spectra (XAS) recorded with either opposite helicity or magnetization direction. XAS spectra were collected on **1**–**3** at the iridium *L*_2,3_ edges under an external magnetic field of *μ*_0_*H*=±17 T and at low temperatures (*T*=2.6–2.9 K). Since the measurements were performed on powdered samples, the isotropic *L*_2,3_ XAS spectra could be approximated as (*σ*^+^+*σ*^−^)/2, where *σ*^+^(*σ*^−^) is the absorption cross-section obtained with helicity and magnetization aligned either parallel (+) or antiparallel (–) (Fig. 3). The XAS spectra are dominated by strong resonance peaks (‘white lines') at both the *L*_3_ (2p_3/2_→5d_3/2,5/2_) and *L*_2_ (2p_1/2_→5d_3/2_) edges with the *L*_3_ edge being significantly more intense.

The spectra of **1** and **2** are virtually identical, whereas for **3**, a slight shift of the white line peak of *ca.* 2 eV towards lower energies is observed concomitantly with an additional component at *ca.* 12 eV higher energy (Supplementary Fig. 9). The latter feature is likely the signature of excitations into delocalized states originating from ligand to metal charge transfer that is not observed in **1** and **2** due to the more ionic Ir–F bond. The effect of SO coupling on the 5d states was quantified through the so-called SO sum rule^21^. It relates the branching ratio (BR) of the white line integrals at the SO split absorption edges to the expectation value of the angular part of the ground state SO operator per hole. For the p→d transitions (*L*_2_ and *L*_3_ edges), the branching ratio can be expressed as


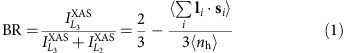


where, 〈*n*_h_〉=〈*n*_h_^*j*=3/2^〉+〈*n*_h_^*j*=5/2^〉 is the total number of holes in the 5d levels and 〈∑_*i*_ **l**_*i*_·**s**_*i*_〉/*ħ*^2^=–3/2 × 〈*n*_e_^*j*=3/2^〉+〈*n*_e_^*j*=5/2^〉 is the expectation value of the one-electron **l**_*i*_·**s**_*i*_ operator summed over all electrons; 〈*n*_e_〉 being the occupation number of the corresponding levels. The white line integrals are larger in **3** than in **1** or **2**, thus more holes in the 5d band should be present for **3** compared with **1** or **2**. If one assumes that **1** and **2** are completely ionic, 〈*n*_h_〉=5, we obtain 〈*n*_h_〉=5.26 for **3**. The corresponding results are shown in Table 1. The quantitative analysis of the XMCD spectra was performed by means of the magneto-optical sum rules providing direct access to *M*_orbital_=–〈*L*_*z*_〉 *μ*_B_ and *M*_spin,eff_=–2 〈*S*_eff_〉 *μ*_B_ (the details of this analysis are given in the ‘Methods' section; Table 1)^22^,^23^. The scaling of the field dependence of the XMCD to the bulk magnetization (inset Fig. 3 and Supplementary Fig. 10; see the ‘Methods' sections) allows the determination of the absolute value of the magnetization at *μ*_0_*H*=+17 T: *M*_total_=1.03, 1.0 and 0.96 *μ*_B_ for **1**, **2** and **3** respectively. From these values, the magnetic dipole contribution, 〈*T*_*z*_〉, and *M*_spin_ (given by *M*_spin,eff_+7〈*T*_*z*_〉) can be estimated (Table 1) without the need of any sophisticated theoretical modelling from *M*_total_=*M*_spin_+*M*_orbital_ considering *M*_spin,eff_ and *M*_orbital_ deduced from the sum rules. The magnetic dipole contribution has never been experimentally determined for any iridate systems, but according to these results, 〈*T*_*z*_〉 cannot be neglected without a significant underestimation of the *M*_orbital_/*M*_spin_ ratio, as previously anticipated^24^.

### Theoretical considerations

**Figure 1:**
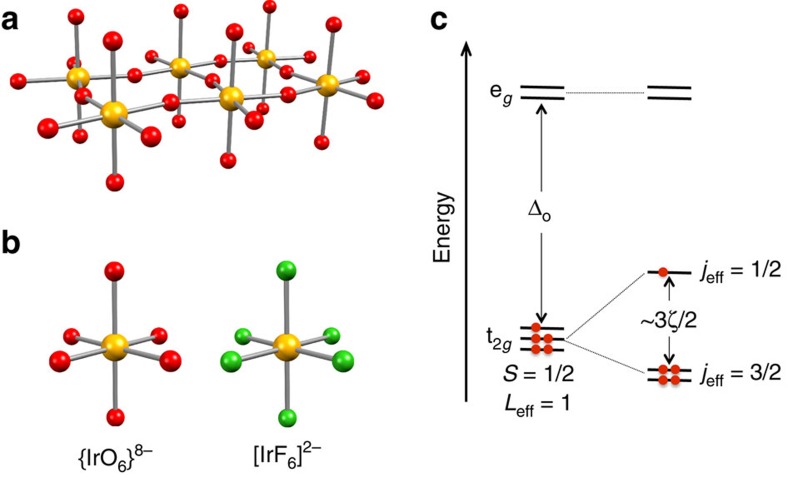
Oxido-iridates versus fluorido-iridates. Ball and stick representations of (**a**) the oxido Ir^IV^-based layer in Sr_2_IrO_4_, (**b**) its smallest {IrO_6_}^8−^ unit and the isoelectronic fluoride counterpart, [IrF_6_]^2−^. (**c**) Energy level diagram for Ir^IV^ (5d^5^; t_2*g*_^5^ electronic configuration). The octahedral ligand field splits the 5d orbitals into e_*g*_ and t_2*g*_ levels, and SO coupling further lifts the degeneracy of the t_2*g*_ levels into filled *j*_eff_=3/2 and half-filled *j*_eff_=1/2 levels. Note that this one-electron picture is equivalent to the SO splitting of the ^2^T_2*g*_ (*O*_h_) term into an *J*_eff_=1/2 ground state and excited *J*_eff_=3/2 state.

**Figure 2:**
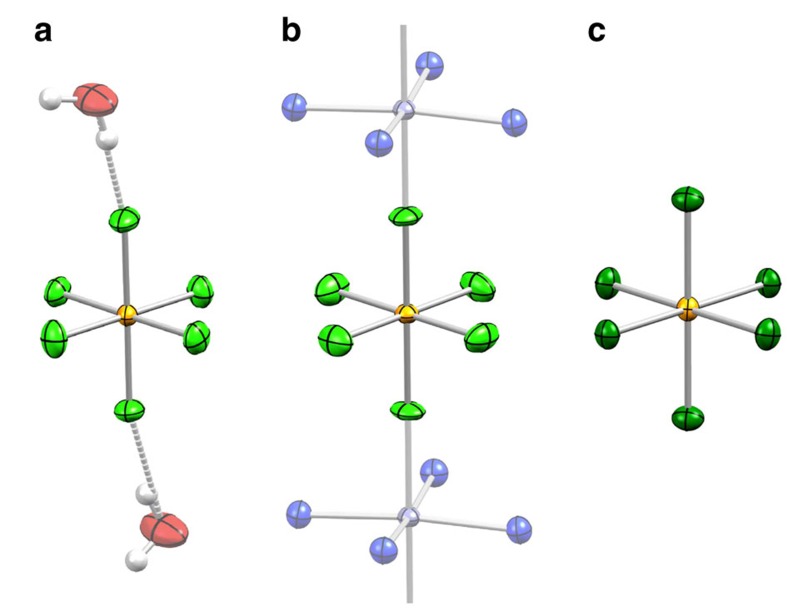
X-ray structure view of the molecular iridates. Thermal ellipsoid plots of **1** (**a**), **2** (**b**) and **3** (**c**) are shown at 80% probability level. The counterions for **1** and **3** and the auxiliary parts of the 1-vinylimidazole ligands in **2** are omitted for clarity. Colour code: Ir, yellow; Zn, grey; Cl, dark green; F, pale green; O, red; N, blue; H, white. Selected bond lengths (Å) and angles (°) for **1**: Ir–F 1.9339(8)–1.9510(8), F–Ir–F_*cis*_ 88.89(4)–91.35(4); for **2**: Ir–F 1.9281(1), 1.9449(1) (only two crystallographically different bond lengths), F–Ir–F_*cis*_ 90, 90.0112(1); for **3**: Ir–Cl 2.3205(3)–2.3370(3), Cl–Ir–Cl_*cis*_ 88.54(1)–91.47(1).

**Figure 3:**
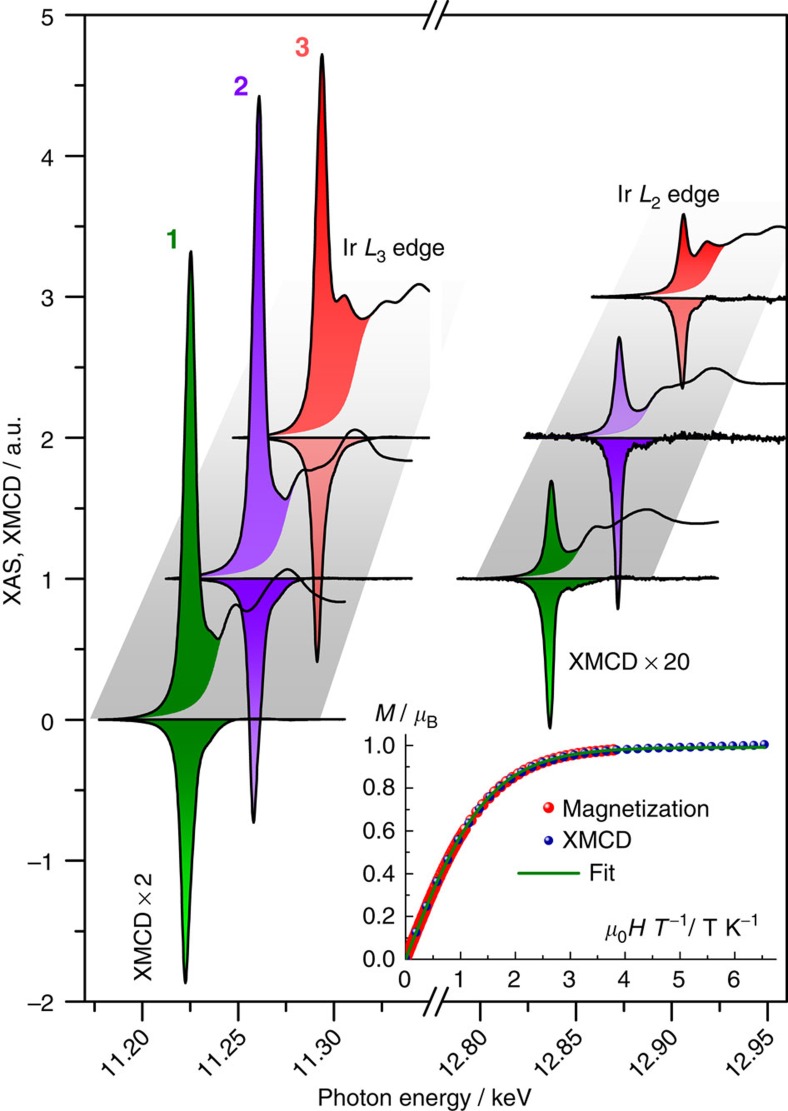
X-ray spectroscopy. X-ray spectra of **1**–**3** showing the isotropic XAS (positive values) and XMCD (negative values) obtained in a magnetic field of +17 T (at 2.7, 2.6 and 2.5 K for **1**, **2** and **3** respectively). The spectra of **2** and **3** were shifted horizontally (35 and 70 eV, respectively) and vertically for clarity. The filled patterns are the integrals used for the sum rule analysis. Inset: Field dependence of the magnetization, *M* versus *μ*_0_*HT*^−1^, of a polycrystalline sample of **2** at *T*=2.0 K. ‘XMCD' designates the field dependence of the XMCD maximum signal at the Ir *L*_3_ edge at *T*=2.6 K. The green line is the best fit of the magnetization and scaled XMCD data to the Brillouin function.

The molecular nature of **1**, **2** and **3** implies that intuitive localized bonding models should be able to disentangle the magnetic moment contributions. The angular overlap model (AOM) allows for a decomposition of the ligand field potential into *σ*- and *π*-bonding parameters^25^. As a first approximation, a cubic [IrF_6_]^2−^ model considering only σ**-bonding (with Δ_*O*_=27,000 cm^–1^, Racah interelectronic repulsion parameters *B*=510 cm^–1^ and *C*/*B*=4.9, and the SO coupling constant *ζ*=3,300 cm^–1^; Fig. 1c)^26^ affords 〈∑_*i*_ **l**_*i*_·**s**_*i*_〉/*ħ*^2^=–2.65, *M*_orbital_=0.74 *μ*_B_ and *M*_spin_=0.36 *μ*_B_, which are close to the ionic values (*M*_orbital_=0.67 *μ*_B_ and *M*_spin_=0.33 *μ*_B_) for a pure *J*_eff_=1/2 system^2^. Whereas the agreement with the experimental results of the fluorido-iridates (Table 1) for 〈∑_*i*_ **l**_*i*_·**s**_*i*_〉/*ħ*^2^ and *M*_orbital_ is surprisingly good, *M*_spin_ is overestimated in this simplistic approach which neglects the covalency. A more realistic model including π-bonding and scaled AOM parameters to account for the different Ir–F bond lengths (see the ‘Methods' section) leads to the same conclusion: 〈∑_*i*_ **l**_*i*_·**s**_*i*_〉/*ħ*^2^=–2.65, *M*_orbital_=0.72 *μ*_B_ and *M*_spin_=0.32 *μ*_B_. However, when covalency is explicitly accounted for by CASSCF calculations, the spin contribution (*M*_spin_=0.22 *μ*_B_) is in much better agreement with the experiment, but the orbital contribution becomes slightly underestimated (*M*_orbital_=0.53 *μ*_B_). Indeed, this departure from cubic symmetry has little influence on the energy of the first excited *J*_eff_=3/2 state (see Fig. 1 caption) as evidenced by calculations (Supplementary Table 2) and near-IR absorption spectroscopy revealing absorption bands from ∼6,000 to ∼8,500 cm^–1^ (Supplementary Fig. 11).

### Electron paramagnetic resonance spectroscopy

For an ideal *J*_eff_=1/2 state, magnetic anisotropy is absent, but minuscule deviations from cubic symmetry may result in strong *g*-factor anisotropy that can be probed experimentally by electron paramagnetic resonance (EPR) spectroscopy. The X-band (*v*=9.634 GHz) EPR spectrum of an [IrF_6_]^2−^ doped Zn(viz)_4_[ZrF_6_] single crystal (∼1% Ir) was measured at 5 K (Supplementary Figs 12–16; Table 2). The experimental eigenvalues of the *g*-tensor are indeed remarkably anisotropic with *g*_*z*_=1.37 and *g*_*xy*_=2.11 in good agreement with the CASSCF calculations leading to *g*_*z*_=1.30 and *g*_*xy*_=2.24 (Supplementary Table 2).

These combined experimental and theoretical results establish that the electronic ground state of the molecular [IrF_6_]^2−^ species is *J*_eff_=1/2 as suggested for the {IrO_6_}^8−^ unit, present in all oxido-iridates.

### Dynamic magnetic properties

**Figure 4:**
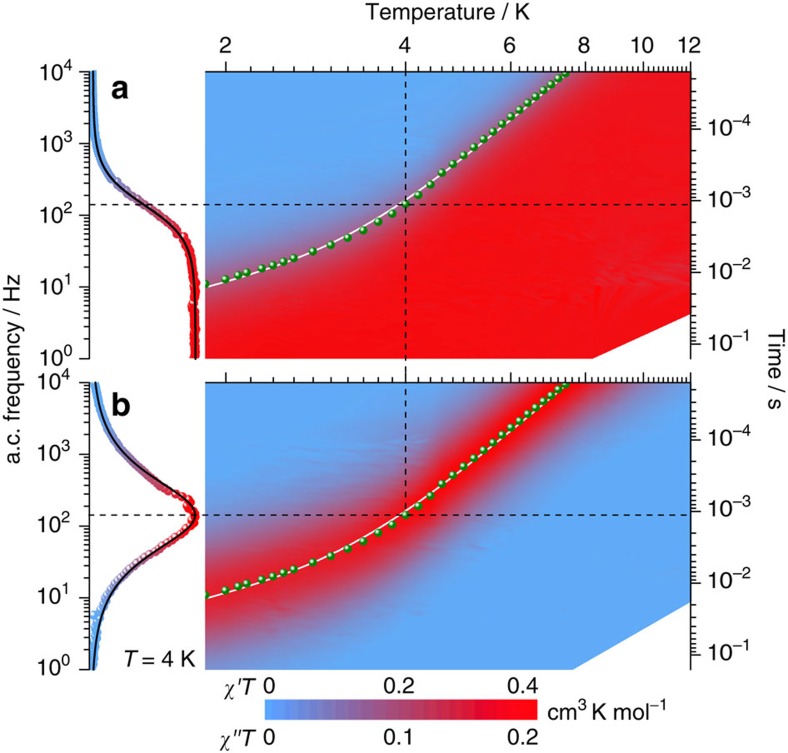
Magnetization dynamics. Two-dimensional frequency/temperature maps of the real (*χ*′*T*, **a**) and imaginary (*χ*″*T*, **b**) components of the a.c. susceptibility-temperature product for a polycrystalline sample of **2** obtained under *μ*_0_*H*=75 mT. Left part: representative 4-K data of the *χ*′*T* and *χ*″*T* frequency dependence with the solid black line being the best fit to the generalized Debye model^27^, that was used for each temperature to determine the relaxation time (*τ*) shown as green dots on the contour plots. The best fit of *τ*^−1^ versus *T* is shown as solid white lines (see text). Dashed lines are guides for the eyes.

The magnetization dynamics of the molecular fluorido-iridates was studied by a.c. susceptibility of **2** at different temperatures below 20 K (Fig. 4 and Supplementary Figs. 17–22 for **1**, **2** and **3**). The presence of peaks in the a.c. frequency (*v*) dependence of the imaginary component, *χ*″*T* (Fig. 4b), which shift with temperature, clearly indicates the slow relaxation of the magnetization, while the vanishing of the real component, *χ*′*T*, in the adiabatic limit (*v*→∞) reveals a blocking of the magnetization that concerns the whole volume of the material. Note that for the three compounds, the slow dynamics of the magnetization is observed only on the application of a small static magnetic field that likely serves to decouple the Ir^IV^ *J*_eff_=1/2 from nuclear spins as justified by a comparable magnitude of the applied field (75 mT) to the width of the EPR spectra (Supplementary Fig. 15).

The real and imaginary components of the a.c. susceptibility could be well-fitted to a generalized Debye model^27^ with small values of the distribution parameter (Supplementary Fig. 23) reflecting a single characteristic relaxation time. The temperature dependence of the extracted relaxation time is shown for **2** in Fig. 4 (and also Supplementary Fig. 24 for **1**–**3**). In these molecular iridates, the spin-lattice relaxation rates *τ*^–1^ were modelled considering Raman and phonon-bottlenecked direct processes, described as a sum of power laws, *τ*^–1^=*CT*^7^+*DT*^2^, and leading to *C*=46 × 10^–3^ s^–1^ K^–7^, *D*=17 s^–1^ K^–2^ for **2** (ref. 28). The magnetization dynamics appears to be similar in **1**, **2** and **3** (Supplementary Fig. 24), suggesting that this property is an intrinsic characteristic of the Ir^IV^ electronic structure. The slow relaxation of the magnetization in **2** was confirmed by muon spin relaxation (*μ*^+^SR) measurements at low temperatures (above 1.9 K; Supplementary Figs 25 and 26), where the implanted muons probe the local dynamics of magnetic fields, thereby ruling out any long-range order. This conclusion is further supported by magnetization measurements on an oriented single crystal of **1** and **2** below 2 K (Supplementary Figs 27–30). The magnetic susceptibility (*χ*=d*M*/d*H*) estimated from these experiments in the zero-field limit follows a Curie–Weiss law, *χ*=*C*/(*T*−*θ*), down to lowest accessible temperature of 0.03 K, confirming the absence of magnetic order (Supplementary Fig. 31). For **2**, the magnetization reaches saturation faster when the d.c. field is applied perpendicular to the *C*_4_ axis rather than along this axis (Supplementary Figs 29 and 30), confirming the easy-plane magnetic anisotropy in agreement with the extracted *g*-factors (*vide supra*). The temperature and magnetic field sweep rate dependence of the magnetization revealed the existence of a weak hysteretic behaviour. At the lowest temperatures (<0.4 K) and in agreement with the a.c. susceptibility data (Fig. 4), butterfly-shaped *M* versus *μ*_0_*H* hysteresis loops for both **1** and **2** (Supplementary Figs 27–30) confirm the presence of phonon-bottlenecked direct processes that dominate the magnetization relaxation^29^.

## Discussion

The principle of dimensional reduction in solid-state structures is based on the formation of a derived compound, A_*na*_MX_*x*+*n*_, from a parent MX_*x*_ precursor and an A_*a*_X salt, thereby forcing termination of M–X–M polymerization^30^. If A is voluminous, for instance an organic cation, child compounds with structurally and magnetically isolated molecular units can be formed. For many oxides, the ultimate dimensional reduction to molecular {MO_*x*_}^*y*−^ is impeded by the progressive development of large localized negative charges (for example, for {IrO_6_}^8−^). Thus, truly single-metal ion analogues of most oxides are difficult, if not impossible, to isolate. In the case of octahedral species, no example has been reported so far. As discussed herein for Ir^IV^, the exchange of oxide with a less negatively charged fluoride results in dimensional reduction of the iridate as compared with the ternary iridium oxide parents. Although their local structures are obviously comparable, the electronic resemblance between these compounds should be discussed here. The branching ratios extracted from XAS (0.85; Table 1) are identical for **1** and **2**, and very close to the values found for oxido-iridates, for example, 0.87 for Sr_2_IrO_4_ and 0.85 for Y_2_Ir_2_O_7_ (refs 31, 32). In addition, the *L*_2_/*L*_3_ XMCD intensity ratio of 4.7% for **2** is almost identical to the value determined for Sr_2_IrO_4_ (∼5%) (ref. 33). This striking agreement demonstrates the resemblance of the electronic structure of the {IrO_6_}^8−^ moieties in oxido-iridates and the molecular [IrF_6_]^2−^ unit. A *M*_orbital_/*M*_spin_=〈*L*_*z*_〉/2〈*S*_*z*_〉 ratio of ∼2.5 in Sr_2_IrO_4_ was recently obtained by non-resonant magnetic X-ray scattering that circumvents the need for the estimation of 〈*T*_*z*_〉 (ref. 34). For comparison, *M*_orbital_/*M*_spin_ ratios of 3.3, 3.3 and 2.1 are obtained for **1**, **2** and **3**, respectively. The slightly larger ratio for **1** and **2** as compared with Sr_2_IrO_4_, and **3** is attributed to the weaker covalency of the Ir–X bond for X=F than for X=O, Cl, as reflected in the nephelauxetic series^35^. It is worth emphasizing that the fluoride ion is the least nephelauxetic of all known ligands, and thus the [IrF_6_]^2−^ moiety has the closest proximity to a perfectly ionic iridate. As a less-pronounced covalency would induce a larger *M*_orbital_/*M*_spin_ ratio^36^, a larger orbital magnetic moment is found in [IrF_6_]^2−^ over {IrO_6_}^8−^ and [IrCl_6_]^2−^, as expected.

In oxido-iridates, the purity of the *J*_eff_=1/2 state depends crucially on the structural deviation from the octahedral symmetry, rendering oxido-iridate systems with weakly distorted {IrO_6_}^8−^ octahedra highly interesting^37^. Remarkably, the [IrF_6_]^2−^ moiety in **2** is structurally closer to cubic than any reported oxido-iridate system. Therefore fluorido-iridates are promising materials for stabilizing an ideal *J*_eff_=1/2 Mott ground state as was already concluded from the experimentally determined values of *M*_orbital_ and *M*_spin_. Paramagnetic relaxation in oxido-iridates has been reported for diamagnetically doped honeycomb systems where the imperfect stoichiometry resulted in the blocking of the magnetization at low temperature^38^,^39^. Our low-temperature experiments reveal that the slow dynamics of the magnetization is indeed intrinsic to the Ir^IV^ centre, that might have implications for quantum magnetism.

Herein, we have established that a molecular [IrF_6_]^2−^ species and the {IrO_6_}^8−^ unit, present in all oxido-iridates, possess the same electronic *J*_eff_=1/2 ground state. The dominating role of the SO coupling on the peculiar magnetism intrinsic to Ir^IV^ was elucidated by studying spatially isolated, magnetically decoupled, [IrF_6_]^2−^ ions in solids. Whereas strongly perturbed by long-range magnetic interactions in iridates, the 5d spin and orbital moments, as well as their local anisotropy and dynamics have been quantified. These experimental results provide a solid basis for building a realistic model to describe the unusual physics discovered in oxido-iridates. Moreover, as illustrated by the one-dimensional Zn(viz)_4_[IrF_6_] system (**2**), the [IrF_6_]^2−^ species appears to be a robust building-block for the construction of molecule-based architectures. It offers an unprecedented synthetic strategy using the versatile tools of soft chemistry to engineer new materials with exotic properties anticipated to be exclusively reserved for oxido-iridates.

## Methods

### Synthetic methods

Na_2_[IrCl_6_]·6H_2_O was heated to 150 °C *in vacuo* for 6 h, cooled to room temperature (exp. (calc. for Na_2_[IrCl_6_]) weight loss=19% (19%)) and subsequently heated to 200 °C in a flow reactor under F_2_ gas (10% diluted in Ar) and kept for 6 h. The last step was repeated to improve the crystallinity of the white product (exp. (calc. for Na_2_[IrF_6_]) weight loss=19% (22%)) that was identified as Na_2_[IrF_6_] by powder X-ray diffraction (Supplementary Fig. 32). A solution of Na_2_[IrF_6_] (68 mg, 0.19 mmol) in water (1 ml) was added a solution of PPh_4_Cl (200 mg, 0.53 mmol) in water (3 ml). The resulting solution was left undisturbed for 1 h to produce crystalline **1** (114 mg, 59%). Anal. calcd. (found) for C_48_H_44_F_6_IrP_2_O_2_: C: 56.46% (56.58%), H: 4.34% (4.19%). **1** can reversibly be oxidized to [IrF_6_]^−^ (*E*_1/2_=+0.8 V versus Fc^+^/Fc; Supplementary Fig. 33). The addition of 1-vinylimidazole (0.80 g, 8.5 mmol) to a methanol solution (40 ml) of **1** (100 mg, 0.098 mmol) and Zn(NO_3_)_2_·6H_2_O (100 mg, 0.34 mmol) afforded crystals of **2** after 1 day. Yield: 60 mg (82%). Anal. calcd. (found) for C_20_H_24_F_6_IrN_8_Zn: C: 32.11% (32.36%), H: 3.23% (3.07%), N: 14.98% (14.67%). Complex **3** was obtained from (NH_4_)_2_[IrCl_6_] (203 mg, 0.460 mmol) that was first dissolved in aqueous HCl (0.1 M, 100 ml). The solution was heated to 50 °C and filtered hot. To this solution, a solution of PPh_4_Cl (360 mg, 0.963 mmol) in aqueous HCl (0.1 M, 70 ml) was added slowly, inducing an immediate precipitation of orange-brown crystals. On complete addition, the reaction mixture was cooled to room temperature and stirred for 30 min leaving only a slightly coloured solution. The product was filtered off and washed three times with water and dried in a dynamic vacuum. Yield: 453 mg (91%). Anal. calcd. (found) for C_48_H_40_Cl_6_IrP_2_: C: 53.20% (53.24%), H: 3.72% (3.67%).

### Crystallography

Powder X-ray diffraction patterns of Na_2_[IrF_6_] were obtained on samples mounted under nitrogen in an air-tight aluminium alloy cell and collected on a PANanalytical Bragg–Brentano *θ*-2*θ* geometry diffractometer (Cu K*α* radiation) equipped with a secondary monochromator over an angular 2*θ* range of 5–80°. Rietveld refinement of the data was performed in the TOPAS (version 4) program^40^. Atomic positions were allowed to refine freely without restraints, while thermal parameters for all atoms were fixed. A suitable fit (*R*_wp_=13.46%) was obtained using a structural model similar to that of Na_2_[GeF_6_] (ref. 41), with an expanded unit cell (space group *P*321, *a*=*b*=9.32858(24) Å, *c*=5.13417(19) Å, *α*=*β*=90°, *γ*=120°; Supplementary Fig. 32). Single-crystal X-ray diffraction studies on **1**–**3** (Supplementary Table 1) were performed at 122(1) K on a Bruker D8 VENTURE diffractometer equipped with Mo K*α* high-brilliance I*μ*S radiation (*λ*=0.71073 Å), a multilayer X-ray mirror, a PHOTON 100 CMOS detector, and an Oxford Cryosystems low-temperature device. The instrument was controlled with the APEX2 software package. The structures were solved in Olex2 using the olex2.solve structure solution program (Charge Flipping) and refined using the olex2.refine program^42^. All non-hydrogen atoms were refined anisotropically and hydrogen atoms were placed at calculated positions and refined as riding atoms with isotropic displacement parameters.

### Magnetometry

The magnetic measurements were carried out between 1.8 and 280 K with applied d.c. fields ranging from –9 to +9 T, with the use of a MPMS-XL Quantum Design SQUID magnetometer and a PPMS-9 Quantum Design susceptometer. Measurements were performed on polycrystalline samples sealed in polyethylene bags (typically 3 × 0.5 × 0.02 cm; 15–30 mg) and immobilized in mineral oil. a.c. susceptibility measurements were performed with an oscillating field of 1–6 Oe with a frequency from 1 to 10,000 Hz. Before the experiments, the field-dependent magnetization was measured at 100 K to confirm the absence of any bulk ferromagnetic impurities. The magnetic data were corrected for the diamagnetic contributions from the sample, sample holder and mineral oil. Micro-SQUID magnetic measurements on single crystals were carried out in the field range from –1.4 to +1.4 T and in the 5.0–0.03 K temperature range. The field was applied in any direction of the micro-SQUID plane with precision >0.1° by separately driving three orthogonal coils^43^.

### X-ray spectroscopy

Polarization-dependent XAS spectra at the Ir *L*_2_ and *L*_3_ edges were acquired at the ID12 beam line at the European Synchrotron Radiation Facility (ESRF), Grenoble, France. A helical undulator of the APPLE-II type allowed the circular polarization of incoming X-rays to be changed for each spectrum. The spectra were obtained by using the total fluorescence yield detection mode in magnetic fields up to ±17 T. Transmission detection was additionally used for **2** to check the validity of the self-absorption correction used for all samples, which took into account the chemical composition, the geometry of the experiment and the solid angle of the X-ray fluorescence detector. XMCD spectra were obtained as the difference of consecutive XAS spectra obtained with opposite photon helicities. In addition, the XMCD spectra were systematically recorded in both field directions to ensure the absence of experimental artefacts. The isotropic spectra were obtained as the sum of spectra with right and left circularly polarized X-rays and were normalized between zero before the absorption edge and one above the edge. The position of the step function describing transitions into the continuum was defined by the following procedure. The maximum of the *L*_3_ white line was assigned to zero energy and the *L*_2_ edge spectrum was shifted in energy to have the first EXAFS oscillations perfectly overlapping for the two spectra. The step function was placed by visual comparison of the shifted spectra. It was systematically checked that shifting the energy of the inflection point of the step function around the chosen position (±2 eV) was not significantly altering the white line integrals. A step function broadening of 6 eV was used, but this parameter does not affect the integrated values of the white line intensities. The white line integral at the *L*_2_ edge was divided by 2.15 reflecting factors of (i) 2 from the occupation ratios of 2p_3/2_ and 2p_1/2_ core states and (ii) 0.15 from the fact that the sum rules are applied to the line intensities *I*(*ħω*) (where *ħω* is the photon energy) and not to the absorption cross sections, *μ*(*ħω*), which are related by *I*(*ħω*)=1/(4*π*^2^*α ħω*) × *μ*(*ħω*) (binding energies for *L*_3_ and *L*_2_ edges are 11.225 and 12.835 keV, respectively). The same normalization procedure was applied to the corresponding XMCD integrals. The precise sample temperature of 2.6–2.9 K and the absolute magnitude of the magnetic moment at *μ*_0_*H*=±17 T was determined by scaling the field and temperature dependent XMCD signal intensity to coincide with the *M* versus *μ*_0_*HT*^−1^ master curve obtained from bulk magnetometry. The normalized spectra were analysed using the magneto-optical sum rules given by equation 2 for *L*_2_ and *L*_3_ absorption edges^22^:


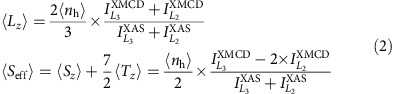


### Ligand field and *ab initio* calculations

The AOM calculations were performed using the Ligfield programme on the full d^5^ configuration^44^,^45^. All matrices were constructed in the weak-field basis and subsequently transformed to the eigen-basis corresponding to the parameter vectors given below. The octahedral component of the ligand field, Δ_*O*_=27,000 cm^–1^, would agree with the AOM parameter *e*_*σ*_=9,000 cm^–1^ in the cubic model. In the more realistic model, a *e*_*σ*_/*e*_*π*_ ratio of 5, and a distance dependence of *e*_*σ*_∝*d*_Ir-F_^–5^ and *e*_*π*_∝*d*_Ir-F_^–7^ (where *d*_Ir-F_ is the Ir-F bond length) was assumed (yielding *e*_*σ*_^*z*^=11,904 cm^–1^, *e*_*σ*_^*xy*^=12,457 cm^–1^, *e*_*π*_^*z*^=2,356 cm^–1^ and *e*_*π*_^*xy*^=2,507 cm^–1^). All *ab initio* calculations were performed with the ORCA 3.0 programme package^46^ using ZORA^47^,^48^ for relativistic corrections and the def2-TZVP basis set for the [IrX_6_]^2−^ moieties and the def2-SVP basis set for the 1-vinylimidazole ligands in calculations when requested in the model. State-averaged CASSCF calculations were performed on experimental geometries using Kohn–Sham starting orbitals obtained with the TPSSh functional. Active spaces of CAS(5,3) (Ir t_2*g*_ orbitals—3 doublet CSFs) and CAS(5,5) (Ir *d*-orbitals—12 doublet configuration state functions (CSFs), 6 quartet CSFs, 1 sextet CSF) were used and the SO interaction taken into account through a mean field approximation (Supplementary Table 2)^49^.

### EPR spectroscopy

The spectra were acquired at *T*=5 K on a Bruker Elexsys E500 spectrometer equipped with a Bruker ER 4116 DM dual mode cavity, an EIP 538B frequency counter and an ER035M NMR Gauss-meter, and with a microwave radiation frequency of 9.634 GHz. The spectra were simulated using home-written software considering an effective electronic *J*_eff_ of 1/2 and taking into account the natural isotopic abundance of ^191^Ir (37.3%) and ^193^Ir (62.3%), which have both a nuclear *I*_Ir_=3/2 spin, as well as the ^19^F nuclear spin (*I*_F_=1/2, 100%). The spectra were computed using the following spin Hamiltonian for each iridium, ^191^Ir and ^193^Ir, isotope situated in a tetragonally distorted octahedral environment:


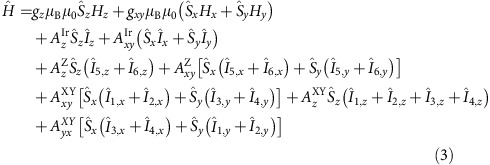


Here, 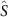 operators refer to the effective spin of *J*_eff_=1/2 pertinent to the electronic ground state. 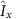, 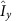 and 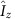 operators refer to the nuclear spin of the iridium nucleus and 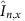, 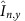, 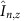 (*n*=1, 2, 3, 4, 5 or 6) refer to the nuclear spin of the fluoride ligands. The fluoride ligands on the positive and negative X-axis are designated with subscripts ‘1' and ‘2', respectively. Similarly, subscripts ‘3' and ‘4', and subscripts ‘5' and ‘6', designate the fluoride ligands on the Y- and Z-axis, respectively. The extracted parameter values are given in Table 2. Remarkably, the estimated hyperfine interactions appear to be almost isotropic, in contrast to the strongly anisotropic superhyperfine interactions to the fluoride ions, which exhibit averaged values on the same order of magnitude as the hyperfine ones. Performing the same experiment on a concentrated crystal of **2** gave virtually identical *g*-factors, but the hyperfine interactions could not be resolved.

### Muon-spin relaxation

The *μ*^+^SR measurements were performed on the Dolly spectrometer at the Paul Scherrer Institut (Villigen, Switzerland). Fully spin-polarized muons were implanted into the sample and acted as a local probe of the internal magnetic fields. The time evolution of the polarization, which was monitored via the anisotropic β-decay of the implanted muons (lifetime 2.2 μs), is determined by the temporal and spatial properties of the local magnetic field^50^. For example, fluctuating magnetic moments in the vicinity of a muon would produce a fluctuating dipolar magnetic field, and therefore, a polarization relaxation from its initial value to zero. The time scale of the relaxation can be extracted from the field dependence of the muon spin-lattice relaxation, *λ*, considering the following equation^51^,


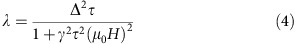


where, Δ is the magnitude of the local magnetic field, *γ* is the gyromagnetic ratio of the muon, *μ*_0_*H* is the applied magnetic field and *τ* is the fluctuation time scale. The equation above assumes that Δ and *τ* are field-independent. From the fit of *λ* as a function of *μ*_0_*H* (Supplementary Fig. 26) *τ*∼0.016 μs is obtained. The small value of *τ* compared with that obtained from a.c. susceptibility measurements is due to the fact that *μ*SR reflects the integrated contribution over *q*-space as a local, point-like probe, while a.c. susceptibility measures the response for *q*=0 (ref. 52).

### Data availability

The X-ray crystallographic coordinates for structures reported in this Article have been deposited at the Cambridge Crystallographic Data Centre (CCDC), under deposition number CCDC 1431835–1431837. These data can be obtained free of charge from The Cambridge Crystallographic Data Centre via www.ccdc.cam.ac.uk/data_request/cif. All other relevant data are available from the authors on request.

## Additional information

**How to cite this article:** Pedersen, K. S. *et al*. Iridates from the molecular side. *Nat. Commun.* 7:12195 doi: 10.1038/ncomms12195 (2016).

## Supplementary Material

Supplementary Information Supplementary Figures 1-33 and Supplementary Tables 1 – 2

Supplementary Data 1 Cif file for compound 1

Supplementary Data 2 Cif file for compound 2

Supplementary Data 3 Cif file for compound 3

## Figures and Tables

**Table 1:** Summary of the X-ray spectroscopy results.

	**1**	**2**	**3**
Branching ratio	0.85	0.85	0.82
〈∑_*i*_ **l**_*i*_·**s**_*i*_〉 (*ħ*^2^)	−2.8	−2.7	−2.5
〈*n*_h_^*j*=5/2^〉/〈*n*_h_^*j*=3/2^〉	4.7	4.4	3.8
*M*_total_ (*μ*_B_)	1.03	1.00	0.96
*M*_spin_ (*μ*_B_)	0.24	0.23	0.31
*M*_orbital_ (*μ*_B_)	0.79	0.77	0.65
〈*T*_*z*_〉 (*μ*_B_)	−0.11	−0.10	−0.081

Obtained with a magnetic field of *μ*_0_*H*=17 T.

**Table 2:** Hyperfine and superhyperfine coupling parameters for [IrF_6_]^2−^ doped in Zn(viz)_4_[ZrF_6_] (∼1% Ir).

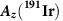	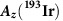	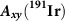	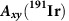	***A***_***z***_^**Z**^	***A***_***xy***_^**Z**^	***A***_***xy***_^**XY**^	***A***_***yx***_^**XY**^	***A***_***z***_^**XY**^
28.6 (12)	31.04 (6)	27.98 (11)	30.68 (7)	66.36 (6)	3.1 (2.5)	98.58 (5)	3.5 (2.7)	10.58 (6)

Parameter values are in units of 10^−4^ cm^−1^.
