# Erratum: Iridates from the molecular side

**DOI:** 10.1038/ncomms12686

**Published:** 2016-08-31

**Authors:** Kasper S. Pedersen, Jesper Bendix, Alain Tressaud, Etienne Durand, Høgni Weihe, Zaher Salman, Thorbjørn J Morsing, Daniel N. Woodruff, Yanhua Lan, Wolfgang Wernsdorfer, Corine Mathonière, Stergios Piligkos, Sophia I. Klokishner, Serghei Ostrovsky, Katharina Ollefs, Fabrice Wilhelm, Andrei Rogalev, Rodolphe Clérac

Nature Communications 7: Article number: 1219510.1038/ncomms12195 (2016); Published: 07 20 2016; Updated: 08 31 2016

The original version of this Article contained an error in which the first affiliation was incorrectly given as ‘CNRS, ICMCB, UPR 9048, Pessac 33600, France'. This has now been corrected in both the PDF and HTML versions of the Article.

