# Supplementary material for: Iridates from the molecular side
Source: Nat Commun. 2016 Jul 20;7:12195. doi: 10.1038/ncomms12195 (PMC4961767; doi:10.1038/ncomms12195)
Supplement: Supplementary Information — Supplementary Figures 1-33 and Supplementary Tables 1 – 2 [file ncomms12195-s1.pdf]

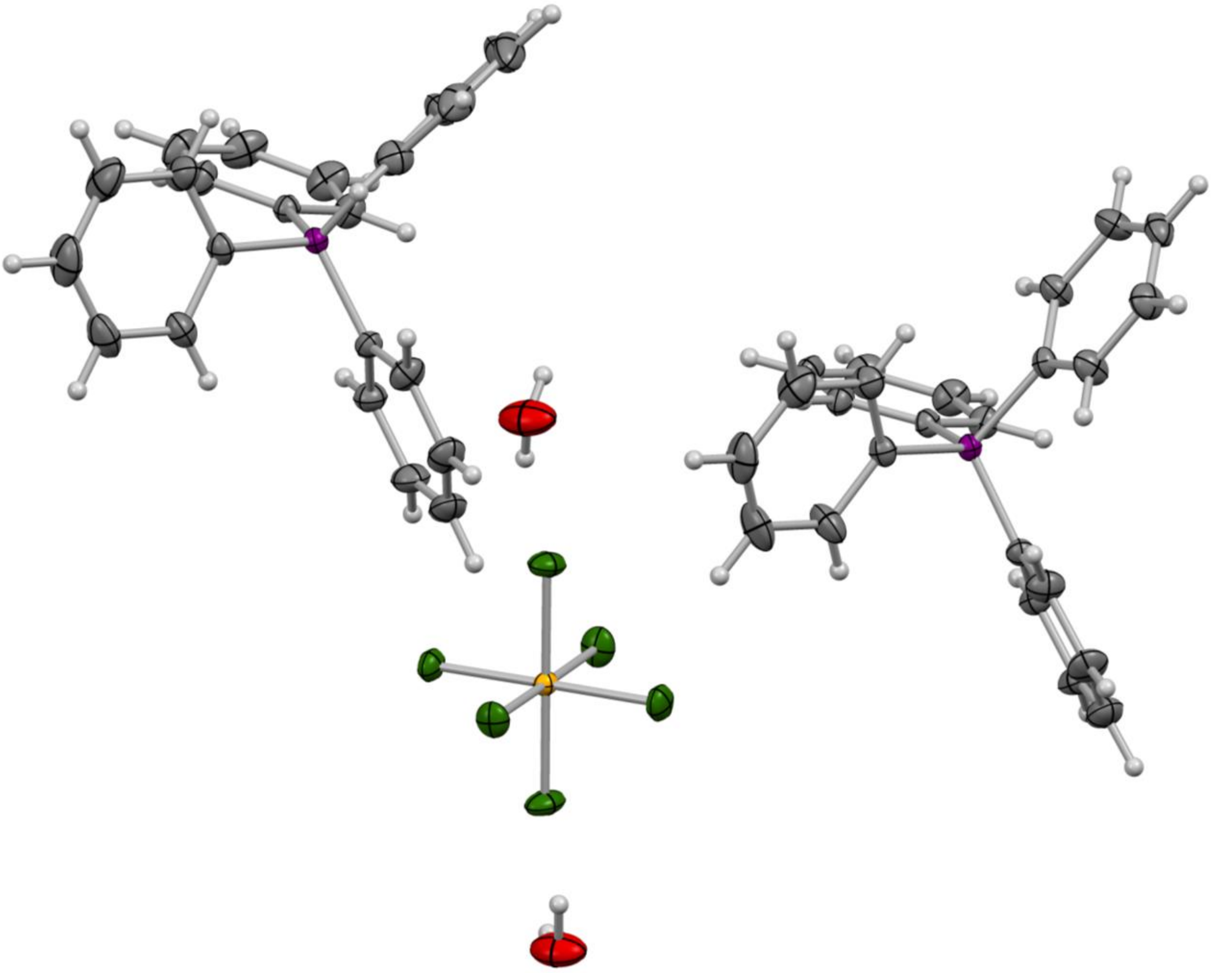

Supplementary Figure 1. Crystal structure of **1** at 122 K (thermal ellipsoids drawn at 70% probability level, except for H atoms shown as spheres). Colour code: Ir, yellow; P, purple; F, green; O, red; C, grey; H, light grey.

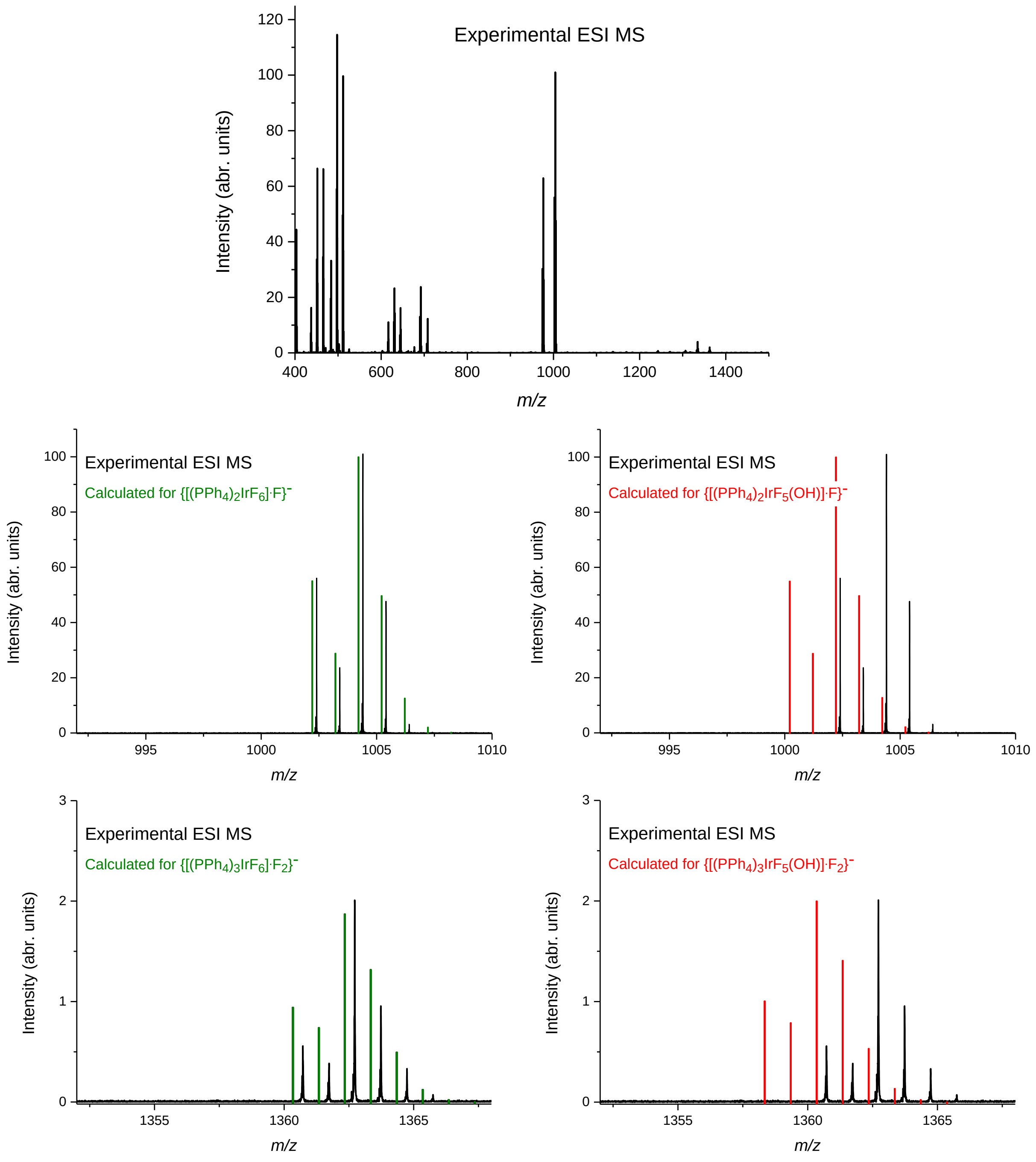

Supplementary Figure 2. Electrospray mass spectra and simulations of **1**. The mass spectra were obtained on a Bruker Solarix XR ESI/MALDI FT-ICR MS instrument using acetonitrile solutions with added formic acid.

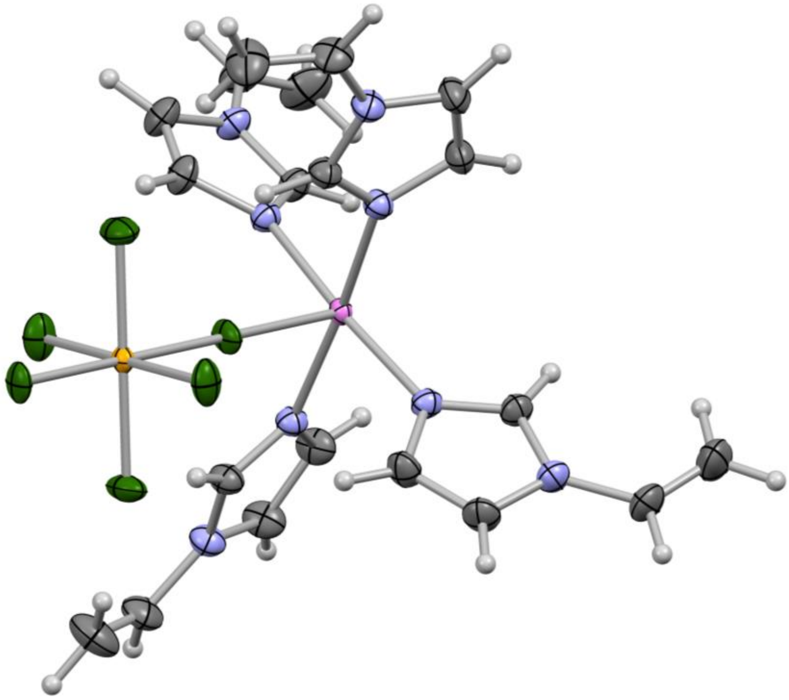

Supplementary Figure 3. Crystal structure of the repeating chain-unit of **2** at 122 K (thermal ellipsoids drawn at 70% probability level, except for H atoms shown as spheres). Colour code: Ir, yellow; Zn, pink; F, green; N, blue; C, grey; H, light grey.

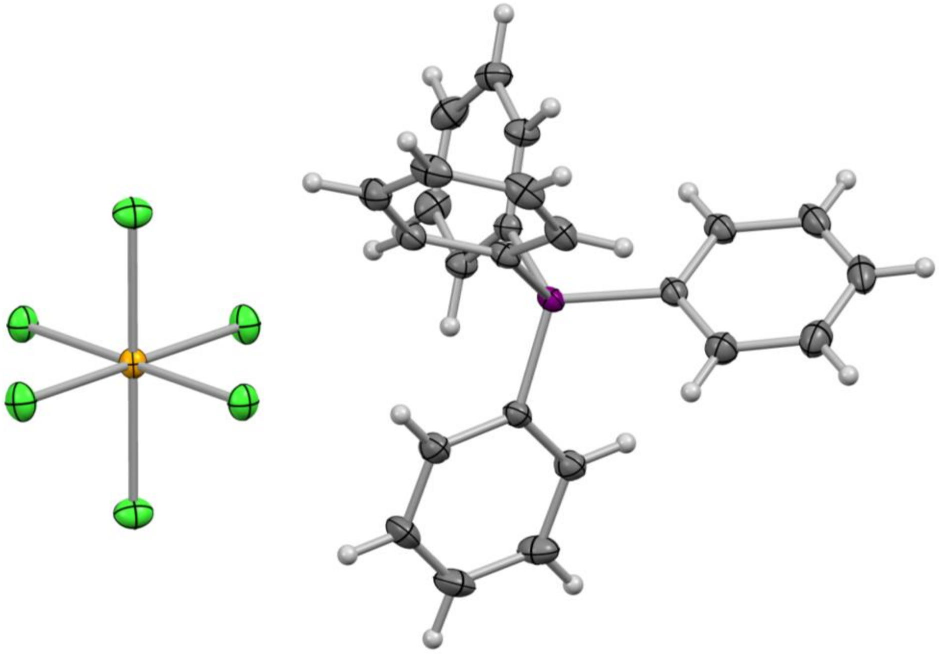

Supplementary Figure 4. Crystal structure of **3** at 122 K (thermal ellipsoids drawn at 70% probability level, except for H atoms shown as spheres). Colour code: Ir, yellow; Cl, light green; P, purple; C, grey; H, light grey.

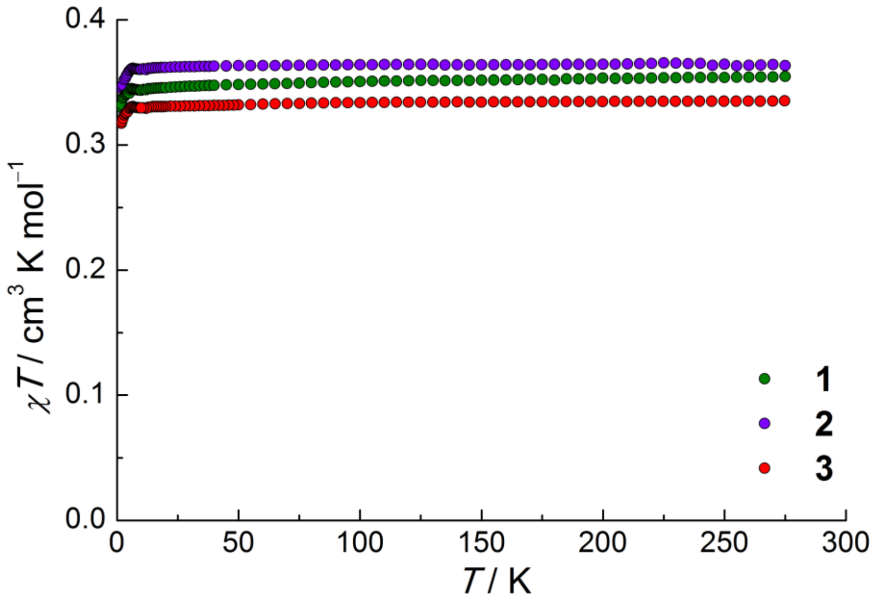

Supplementary Figure 5. Temperature dependence of the susceptibility-temperature product, χT vs. T, of polycrystalline samples of **1–3** obtained with a magnetic field of $\mu_0 H$ = 1.0 T.

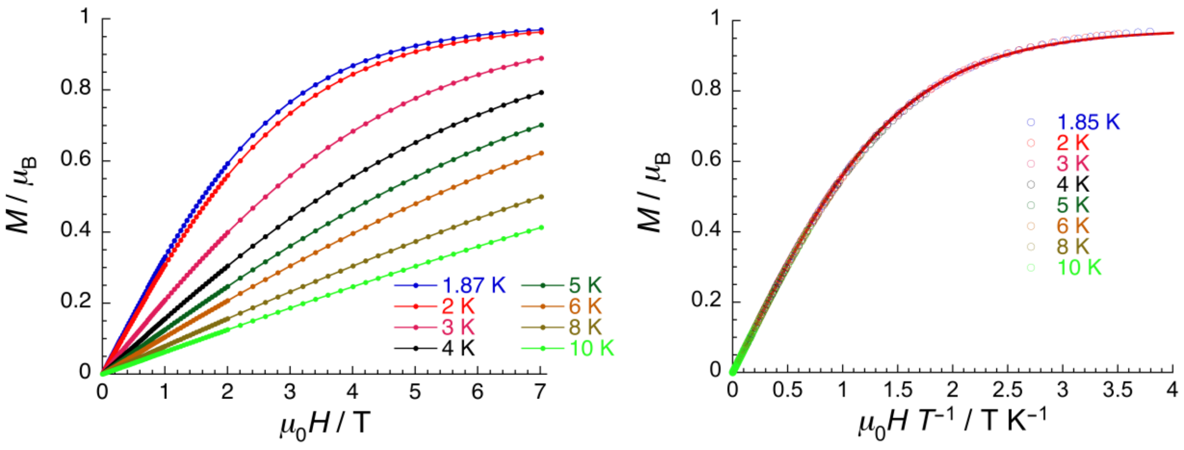

Supplementary Figure 6. Field-dependence of the magnetization obtained on a polycrystalline sample of **1** at selected temperatures. The red solid line (right) is the best fit to the Brillouin function (*g* = 1.95).

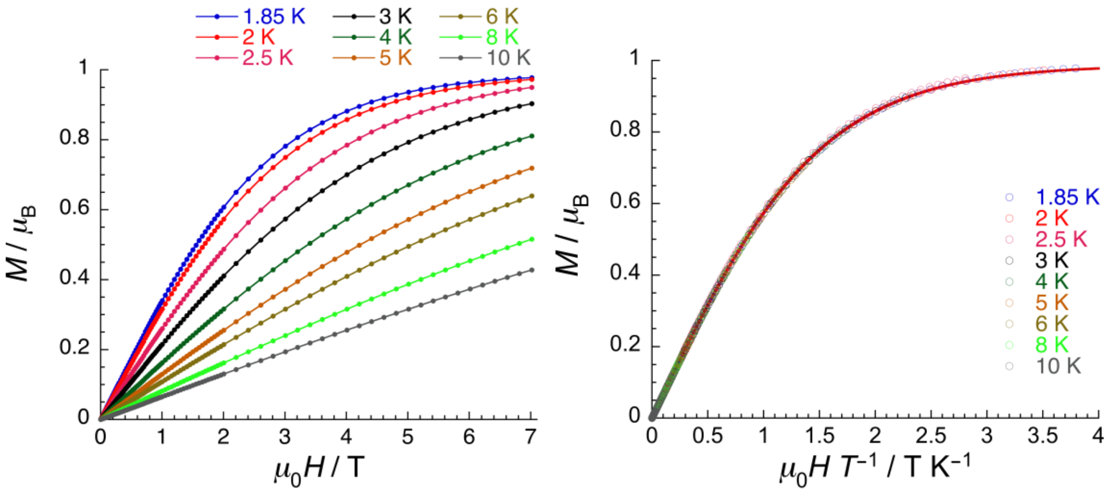

Supplementary Figure 7. Field-dependence of the magnetization obtained on a polycrystalline sample of **2** at selected temperatures. The red solid line (right) is the best fit to the Brillouin function (g = 1.96).

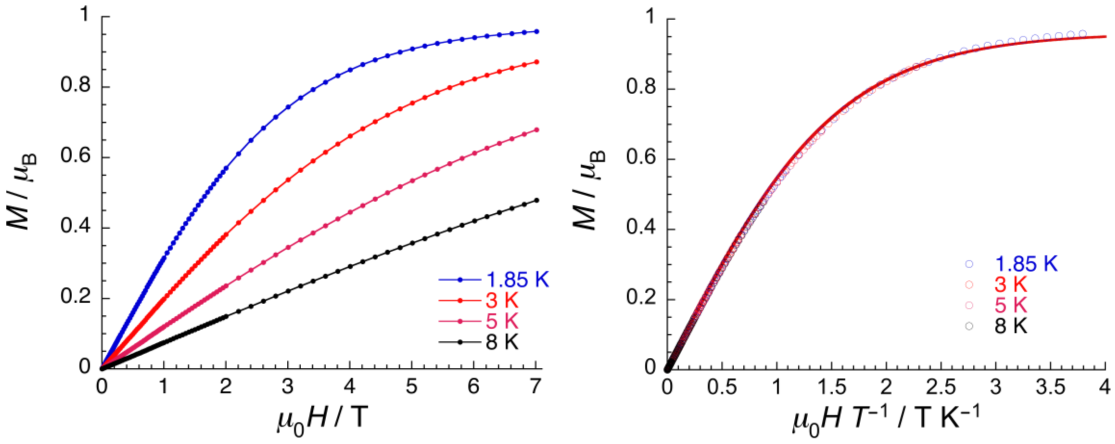

Supplementary Figure 8. Field-dependence of the magnetization obtained on a polycrystalline sample of **3** at selected temperatures. The red solid line (right) is the best fit to the Brillouin function (g = 1.92).

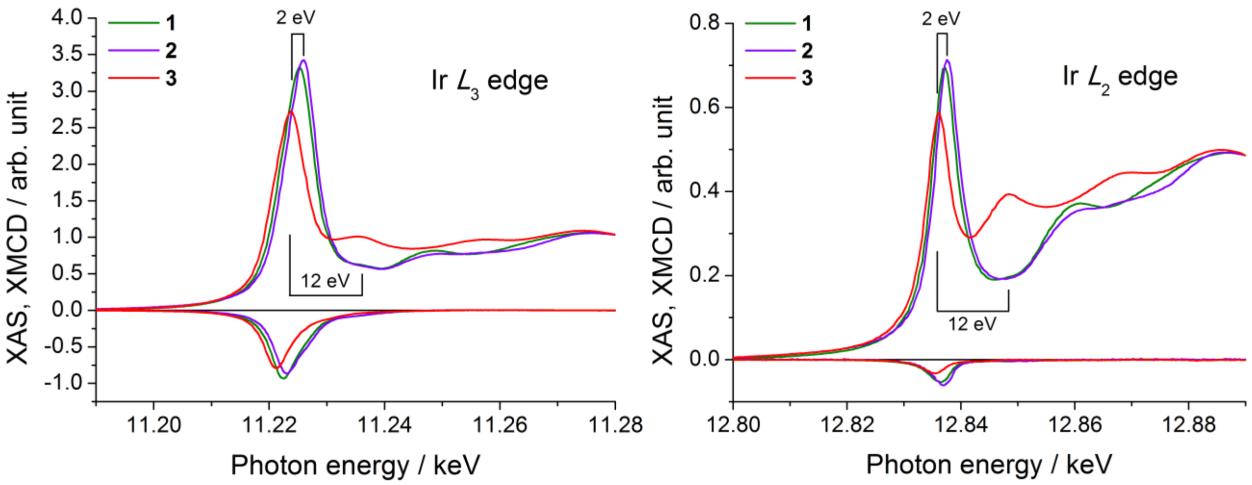

Supplementary Figure 9. Superposed X-ray spectra of **1–3** showing the isotropic XAS (positive values) and XMCD (negative values). The back line defines zero intensity. As discussed in the main text, the shift of the white line peaks of **3** as compared to **1** and **2** by ~2 eV and the additional component at ~12 eV higher energy than the white line in **3** are both indicated.

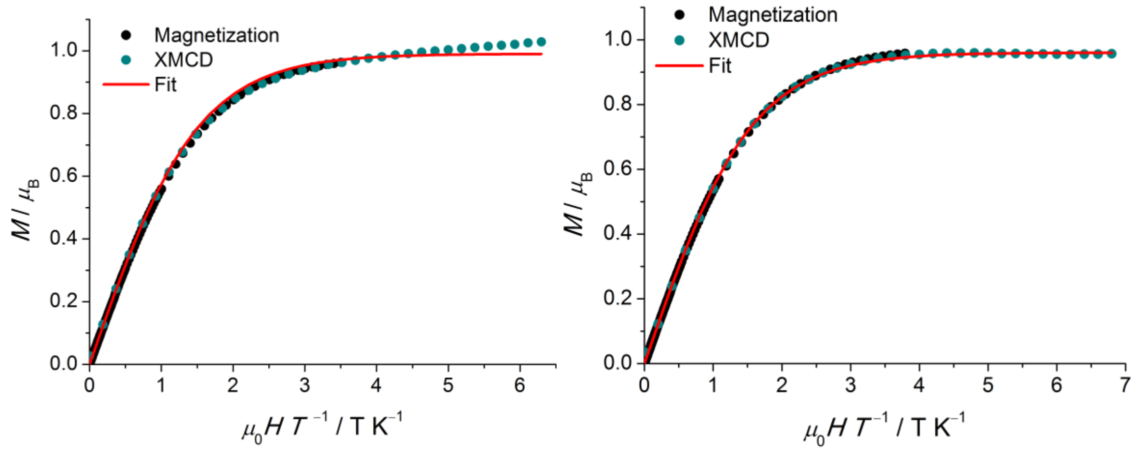

Supplementary Figure 10. Field dependence of the maximum XMCD signal normalized to the bulk magnetization data (Supplementary Figures 6 and 8). The red lines are best fits to the Brillouin function for a J_{eff} = 1/2. Left: **1**, T = 2.7 K, fitted g = 1.96, photon energy = 11221.5 eV. Right: **3**, T = 2.5 K, fitted g = 1.92, photon energy = 11221.3 eV.

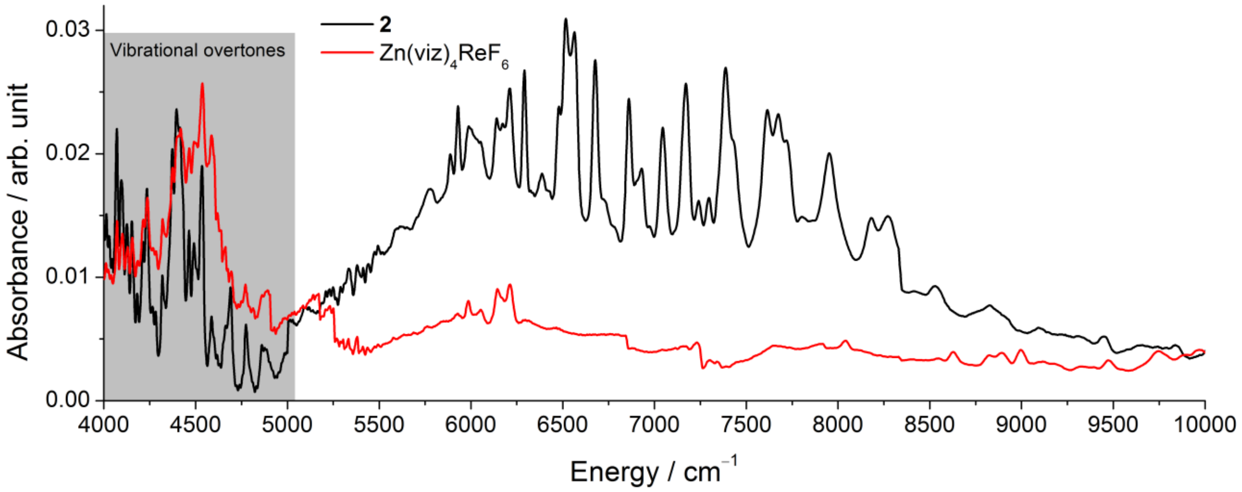

Supplementary Figure 11. Near-infrared absorption spectrum of polycrystalline **2** dispersed between glass plates at 10 K. The red trace is the corresponding absorption spectrum of the isostructural $Zn(viz)_4ReF_6$ compound. The spectra were collected using a Cary 5 spectrophotometer equipped with an Oxford cryostat.

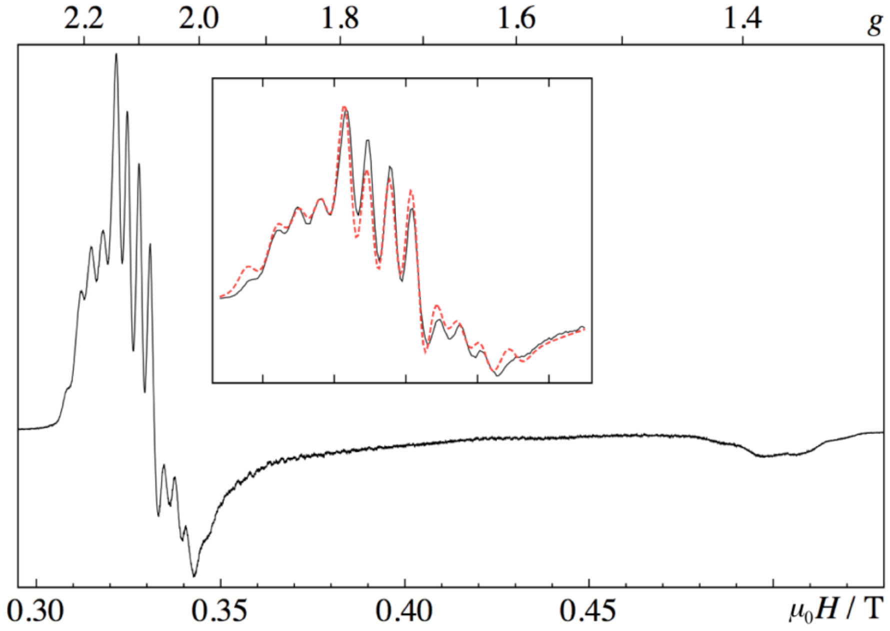

Supplementary Figure 12. EPR spectrum of a powdered sample of [Zn(viz)$_4$][ZrF_6] containing 1% of $[IrF_6]^{2-}$ (ν = 9.643 GHz and T = 5 K). Inset: zoom of the low-field part of the spectrum. The red line is a simulation using the parameters derived from the analysis of the single-crystal spectra given in the main text.

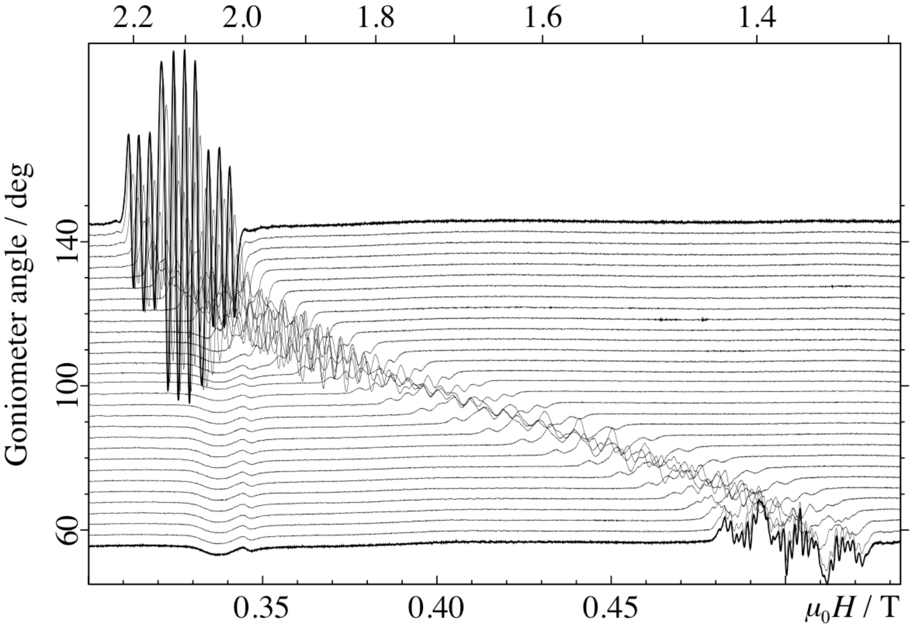

Supplementary Figure 13. Single-crystal EPR spectra of $[Zn(viz)_4][ZrF_6]$ containing 1% of $[IrF_6]^{2-}$ with the magnetic field in the *xz* plane (ν = 9.643 GHz and T = 5 K). The thick-line emphasized the lowest and highest spectra corresponding to the magnetic field being parallel to the *z* and *x* axis, respectively.

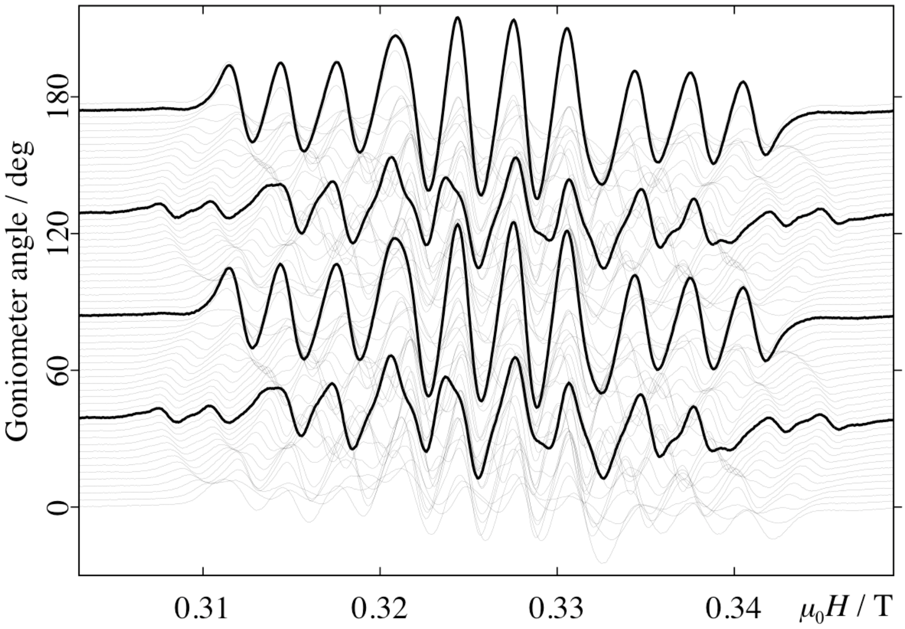

Supplementary Figure 14. Single-crystal EPR spectra of $[Zn(viz)_4][ZrF_6]$ containing 1% of $[IrF_6]^{2-}$ with the magnetic field in the *xy* plane (ν = 9.643 GHz and T = 5 K). The crystal was rotated 3° between each spectrum. Four spectra representing special orientations have been emphasized with thick lines; these are the spectra at goniometer angles of 174° and 84° with the magnetic field along a F-Ir-F bond; similarly, the spectra with the goniometer angles of 129° and 39° correspond to the magnetic field being between two Ir-F bonds.

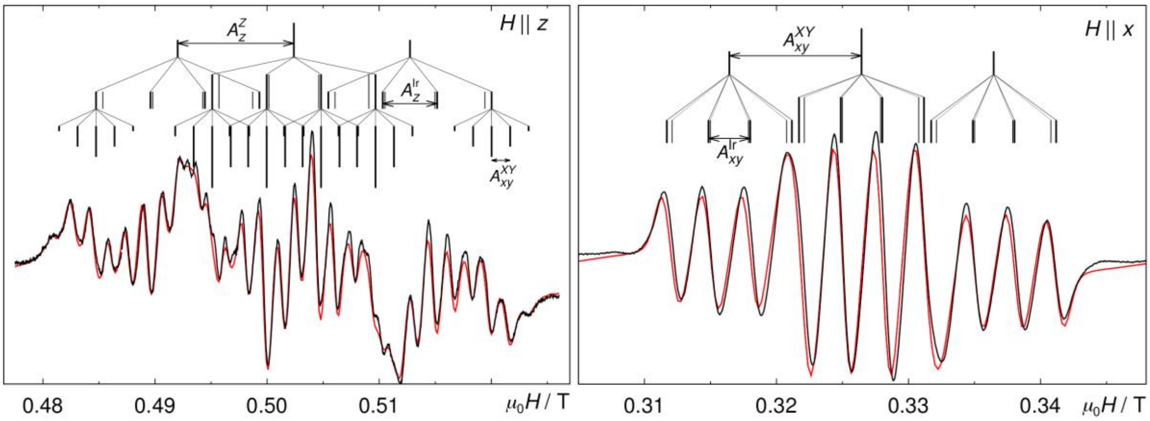

Supplementary Figure 15. Single-crystal EPR spectra of $[Zn(viz)_4][ZrF_6]$ containing 1% of $[IrF_6]^{2-}$ with the magnetic field along the *z* axis (chain direction, left) and along *x* (right) obtained at 5 K (ν = 9.643 GHz). The experimental and calculated spectra are indicated with a black and red line, respectively. The stick diagrams above the spectra identify the iridium hyperfine (A_z^{Ir}, A_{xy}^{Ir}) and fluorine superhyperfine (A_z^Z, A_{xy}^{XY}, A_{xy}^{XY}) interaction terms. For the left part, the lowest part of the stick diagram is drawn only for the ^{193}Ir isotope and only for a selected portion of the spectrum for simplicity.

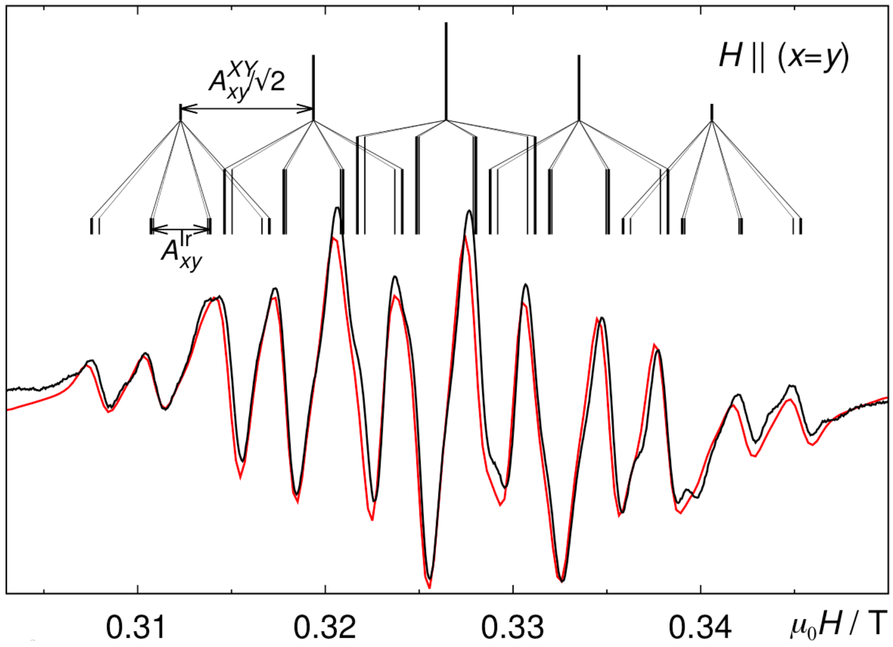

Supplementary Figure 16. Single-crystal EPR spectra of $[Zn(viz)_4][ZrF_6]$ containing 1% of $[IrF_6]^{2-}$ with the magnetic field is directed perpendicular to the *z* axis and in between the *x* and *y* axes (ν = 9.643 GHz and *T* = 5 K). The experimental and calculated spectra are indicated with a black and red line, respectively. The peak-to-peak width of the twelve separate lines are in the 1-1.9 mT interval. The stick diagram identifies the two terms in the spin Hamiltonian (given in the Methods section of the main text) being responsible for the splittings at this orientation of the magnetic field.

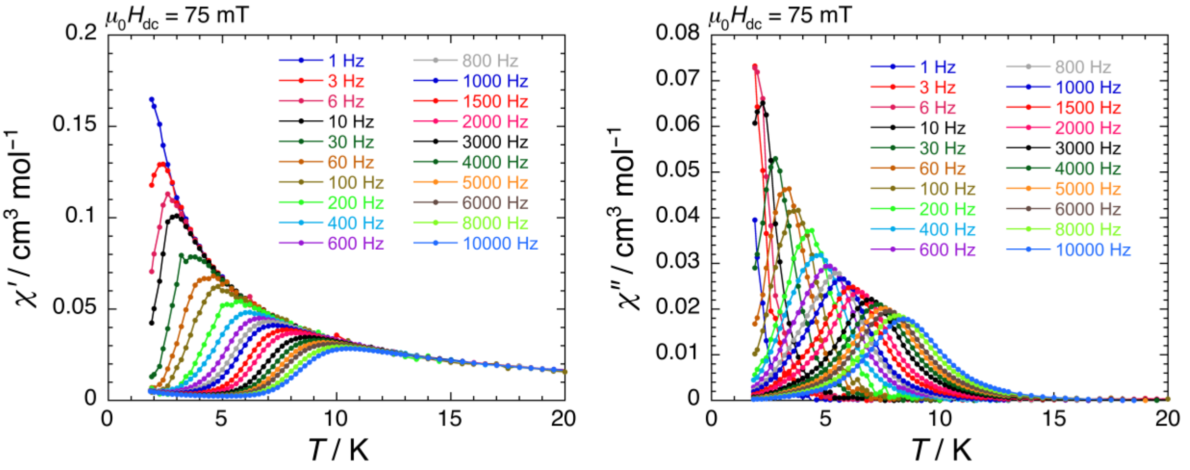

Supplementary Figure 17. Temperature dependence of the ac susceptibility for **1** in a dc field of μ_0H_{dc} = 75 mT.

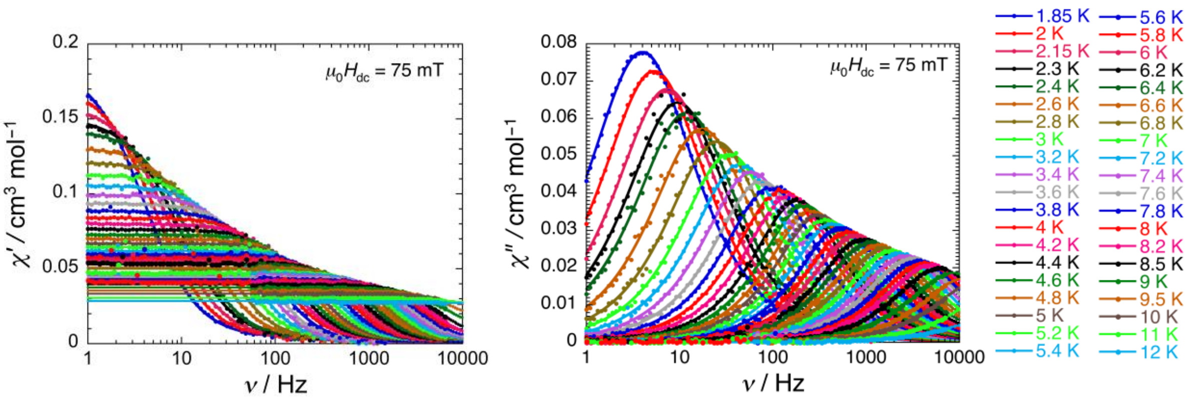

Supplementary Figure 18. Frequency dependence of the ac susceptibility of **1** in a dc field of μ_0H_{dc} = 75 mT.

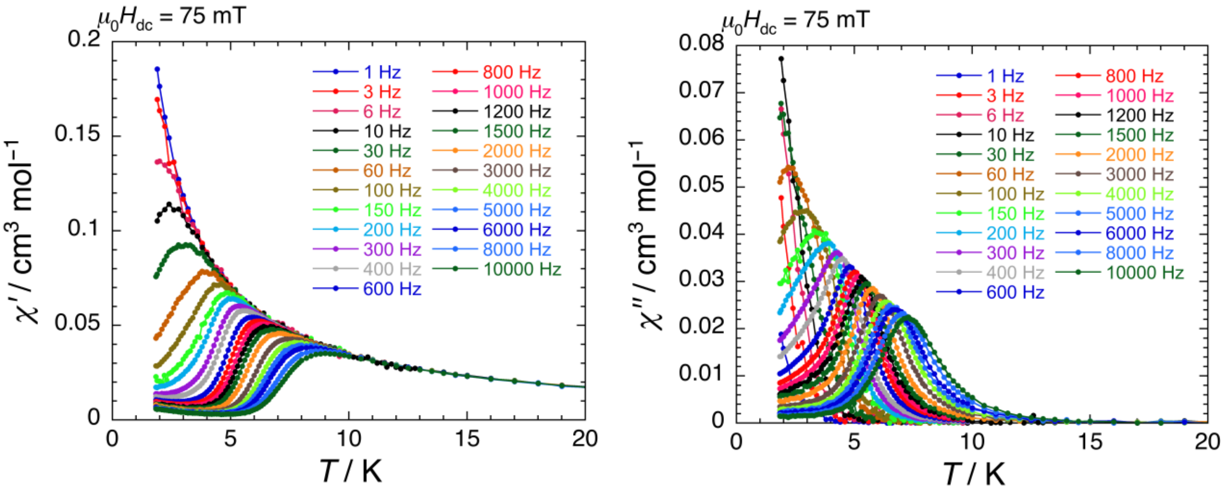

Supplementary Figure 19. Temperature dependence of the ac susceptibility of **2** in a dc field of μ_0H_{dc} = 75 mT.

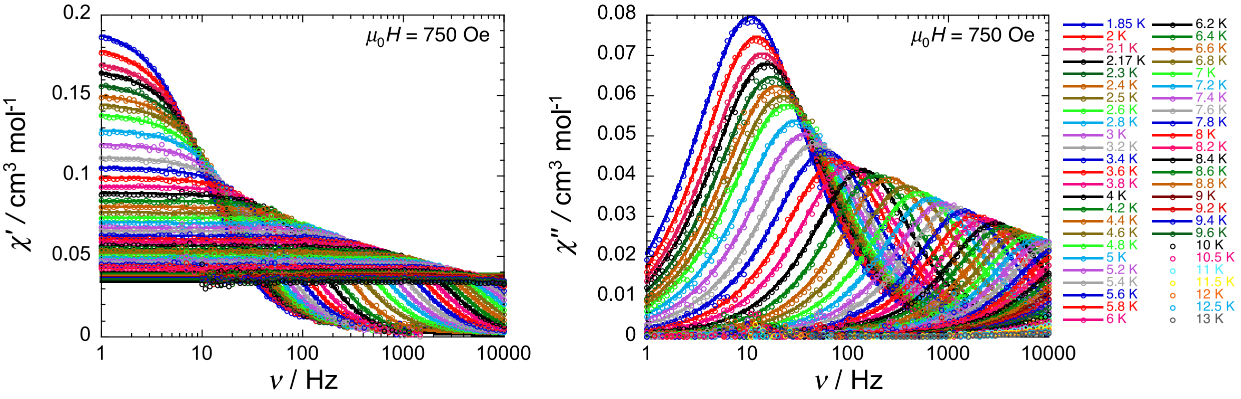

Supplementary Figure 20. Frequency dependence of the ac susceptibility of **2** in a dc field of $\mu_0 H_{dc}$ = 75 mT.

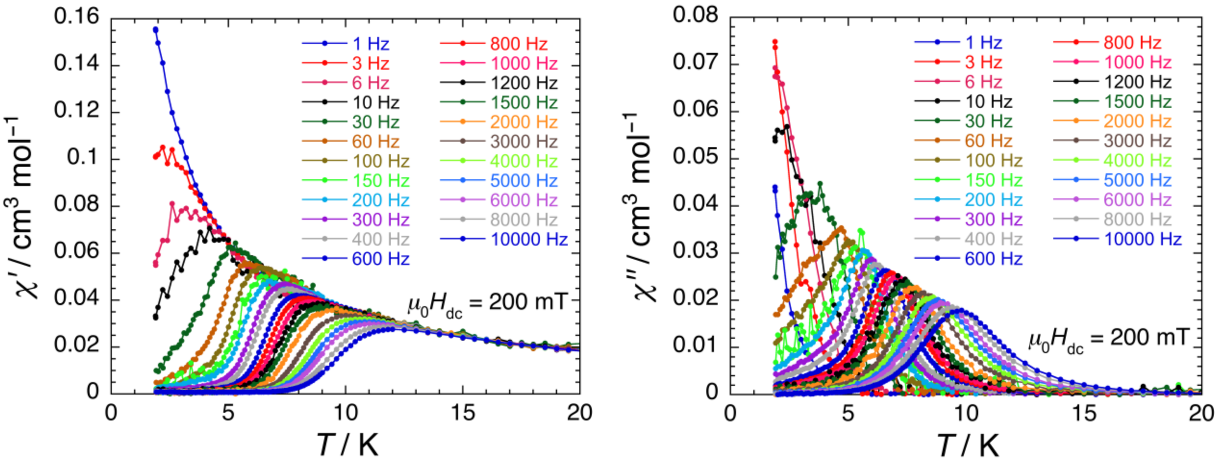

Supplementary Figure 21. Temperature dependence of the ac susceptibility of **3** obtained a dc field of $\mu_0 H_{dc}$ = 200 mT.

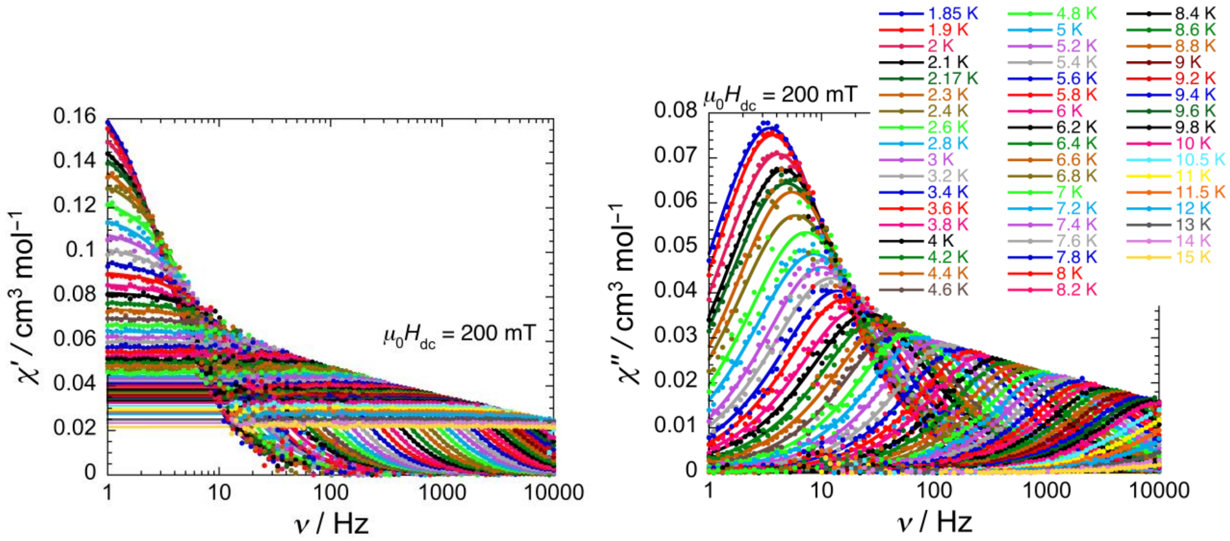

Supplementary Figure 22. Frequency dependence of the ac susceptibility of **3** in a dc field of $\mu_0 H_{dc}$ = 200 mT.

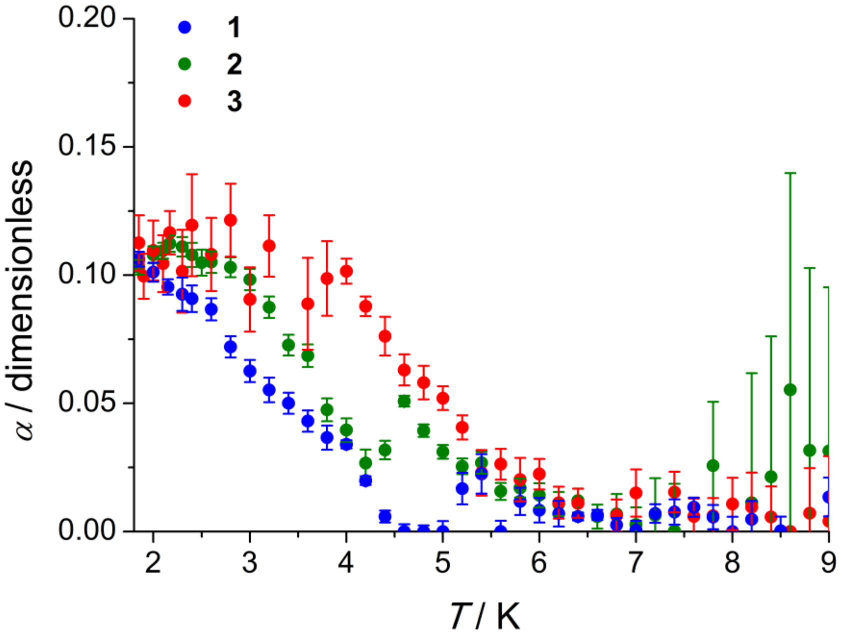

Supplementary Figure 23. Distribution parameter, α, of the generalized Debye model extracted from the fits of $\chi''(\nu)$ (Figures S12, S14 and S16) with its error bar shown as a vertical line.

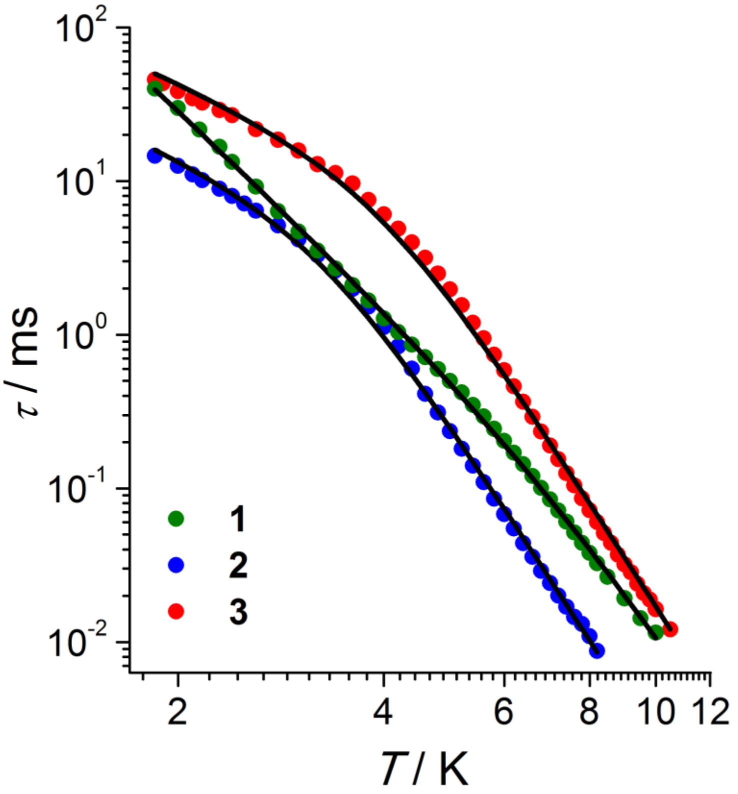

Supplementary Figure 24. Temperature dependence of the relaxation time for **1**, **2** and **3**. The solid lines represent the best fits to the expression $\tau(T)^{-1} = CT^7 + DT^m$. For **2** and **3**, m was fixed to 2, as expected for a phonon-bottlenecked direct process. The best-fit parameters are **1**: $C = 6.6 \times 10^{-3}$ s^{-1} K^{-7}, $D = 1.9$ s^{-1} K$^{-4.2}$, $m = 4.2$; **2**: $C = 46 \times 10^{-3}$ s^{-1} K^{-7}, $D = 17$ s^{-1} K^{-2}. **3**: $C = 5.8 \times 10^{-3}$ s^{-1} K^{-7}, $D = 5.7$ s^{-1} K^{-2}.

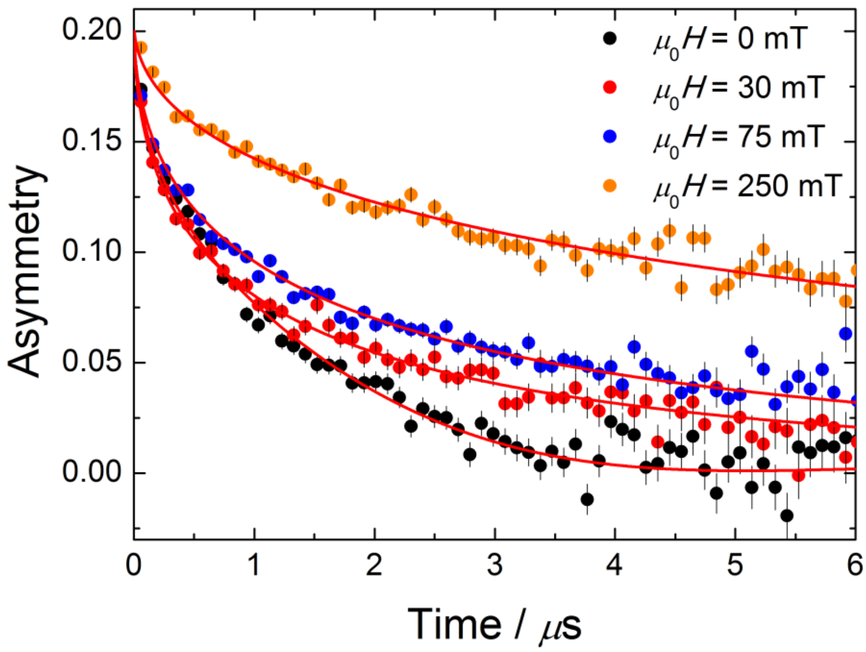

Supplementary Figure 25. Typical muon spin relaxation curves measured in zero and applied magnetic field at T = 1.9 K. The relaxation follows a square root stretched exponential function (solid lines). The field dependence and relaxation form confirm that the local field experienced by the implanted muons is fluctuating.

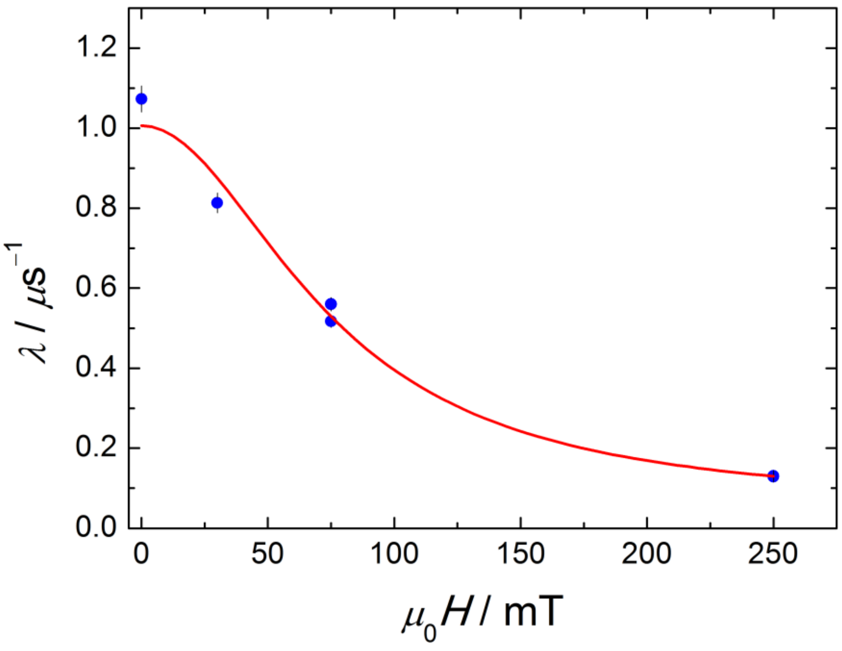

Supplementary Figure 26. The muon spin relaxation rate, λ, as a function of applied field at T = 1.9 K showing a monotonically decreasing value, consistent with the dynamic nature of the internal magnetic fields in the sample. The solid line is a fit to a Lorentzian function as described above.

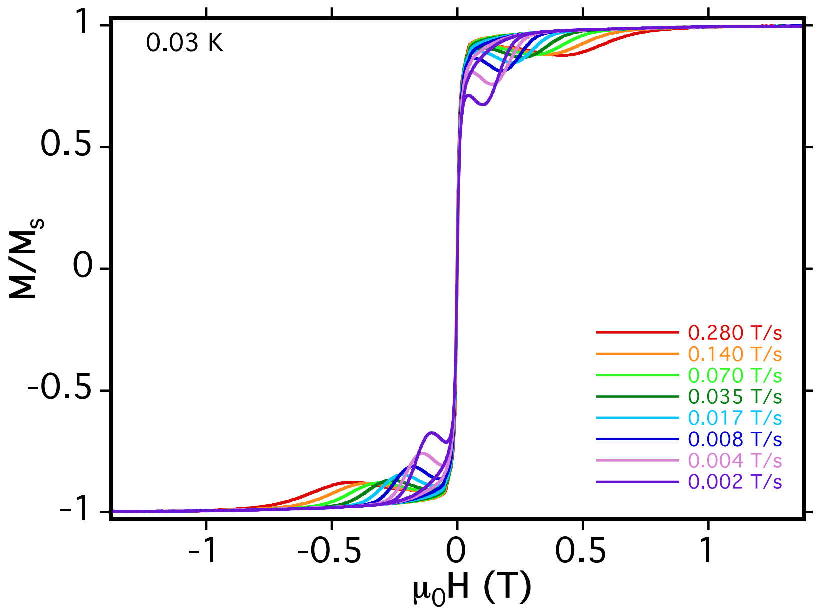

Supplementary Figure 27. Sweep rate dependence at 0.03 K of the magnetization measured on an oriented single-crystal of **1**. The field was aligned parallel to the easy axis of magnetization by using the transverse field method.

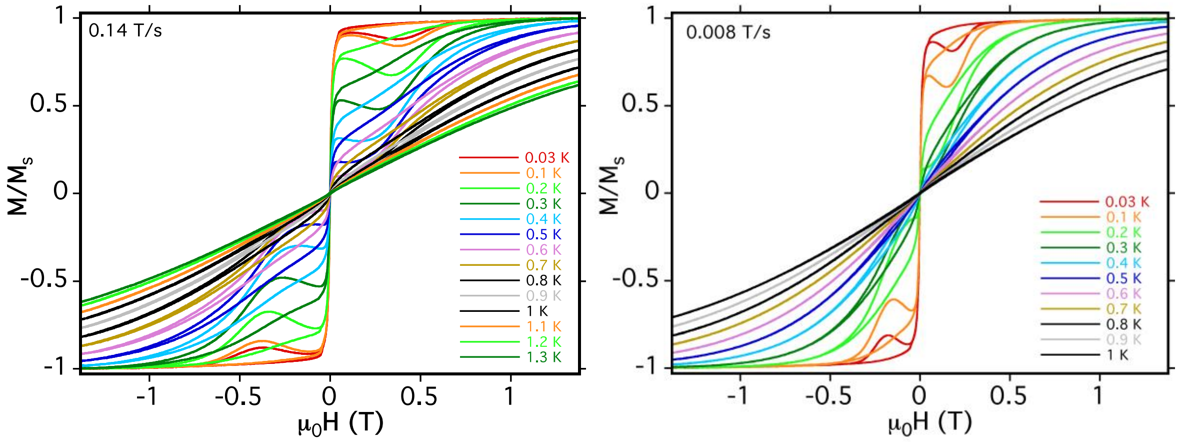

Supplementary Figure 28. Temperature dependence at 0.14 T/s (left) and 0.008 T/s (right) of the magnetization measured on an oriented single-crystal of **1**. The field was aligned parallel to the easy axis of magnetization by using the transverse field method.

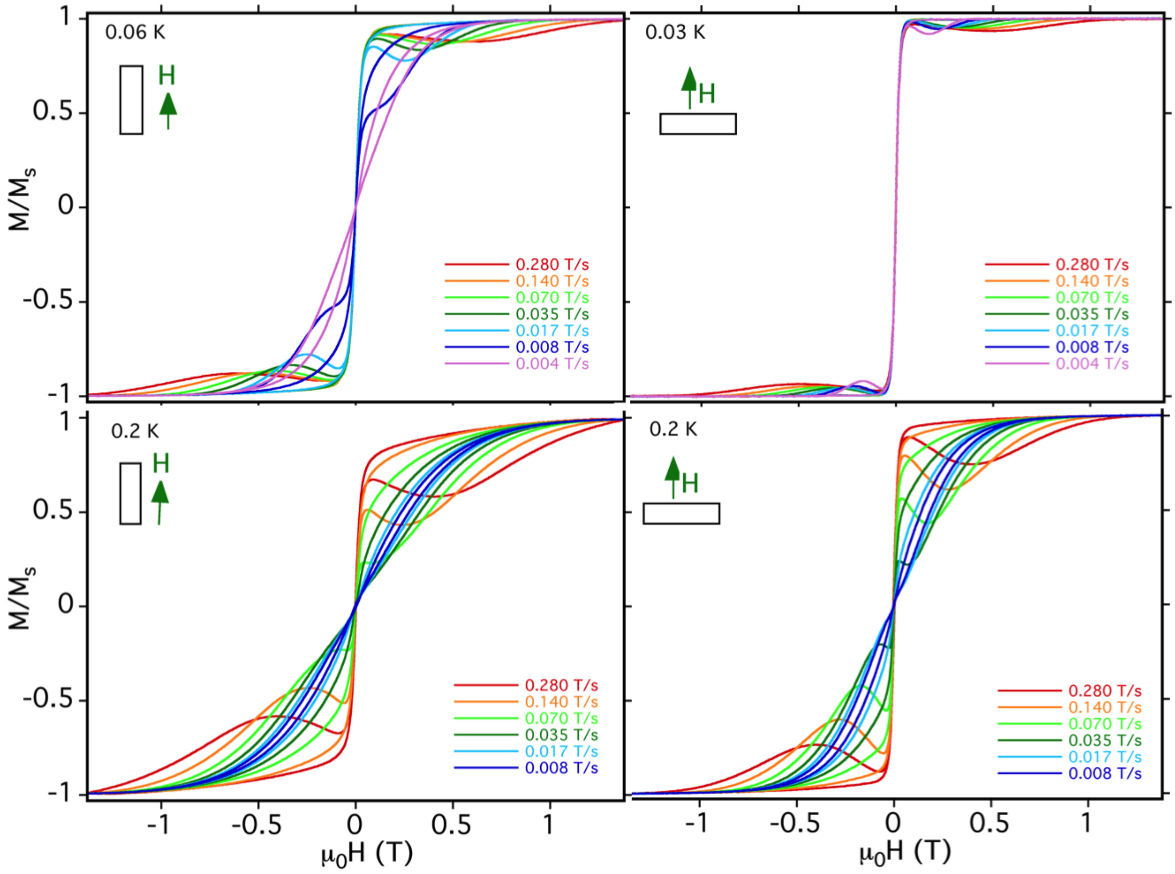

Supplementary Figure 29. Sweep rate dependence of the magnetization measured on an oriented single-crystal of **2** along (left) and perpendicular (right) to the C_4 axis at indicated temperatures.

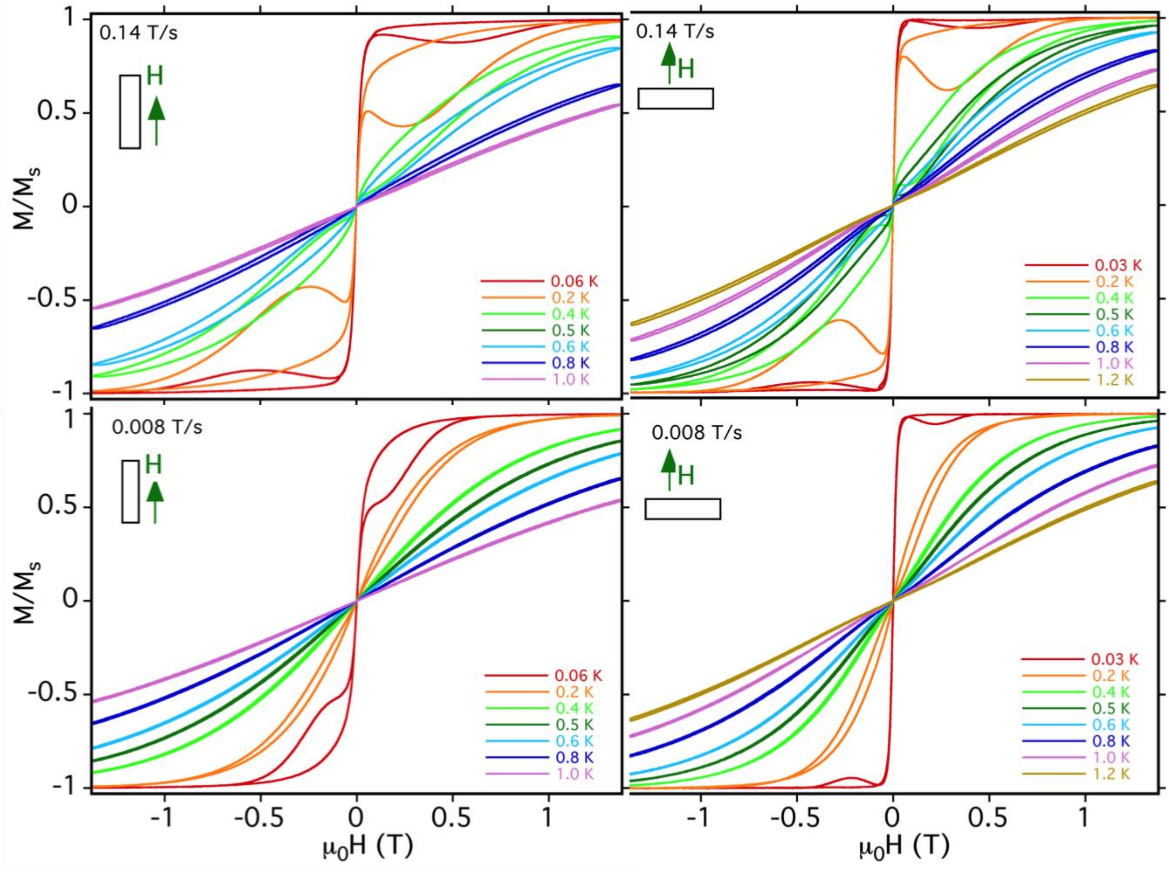

Supplementary Figure 30. Temperature dependence of the magnetization of a single-crystal of **2** along (left) and perpendicular (right) to the C_4 axis at selected sweep rates.

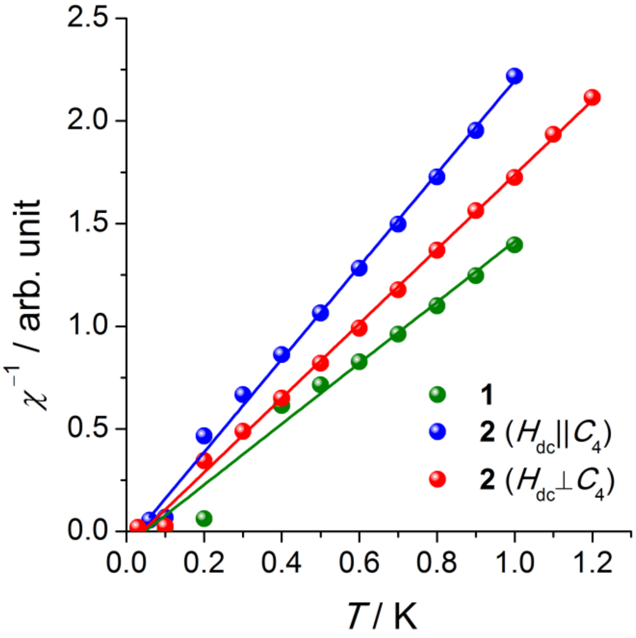

Supplementary Figure 31. Curie-Weiss plots constructed from the temperature dependent data in Figures S20 and S22 at the slowest magnetic field sweeping rate (0.008 T/s). The susceptibility, χ, was determined by the slope of the magnetization curves in the zero-field limit. The fits of the χ^{-1} vs. T data to the Curie-Weiss law $\chi^{-1} = (T - \theta)/C$, are shown as solid lines. The best-fit Weiss constants are θ(**1**) = 46 mK, θ(**2**) = 29 mK and 41 mK when the magnetic field was placed along or perpendicular to the C_4 axis, respectively.

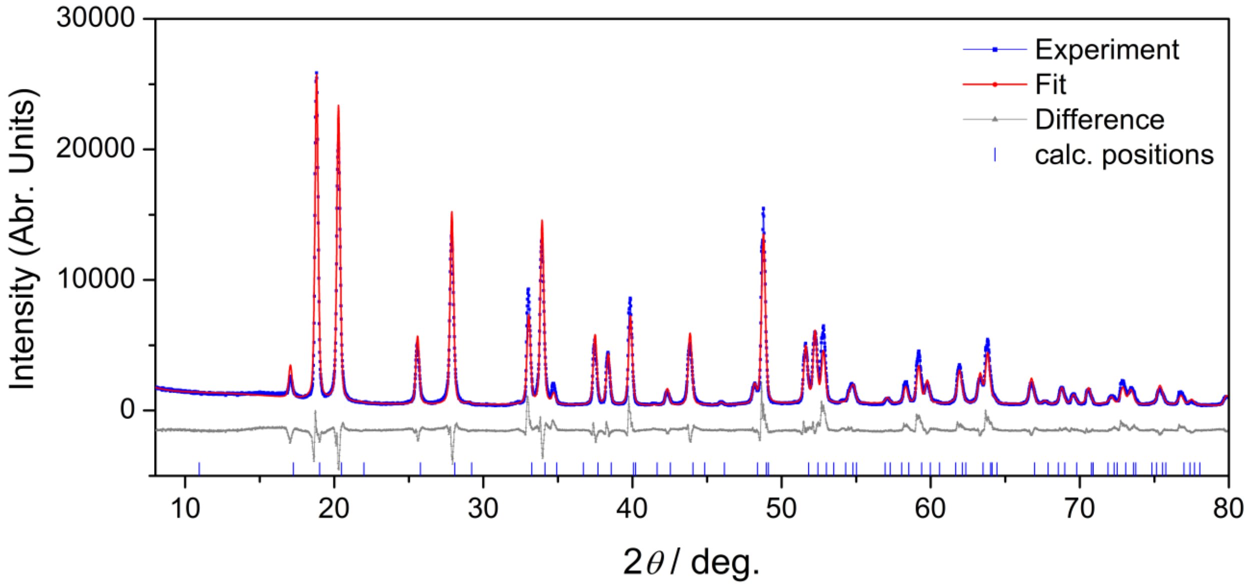

Supplementary Figure 32. Room-temperature powder X-ray diffractogram of $Na_2[IrF_6]$ and its Rietveld refinement.

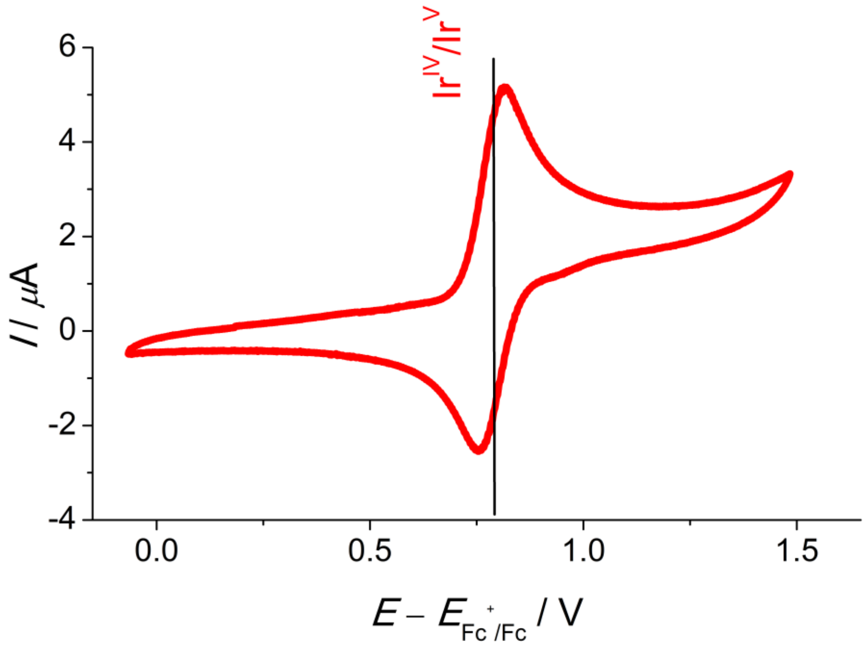

Supplementary Figure 33. Cyclic voltammogram of **1** in MeCN ($[IrF_6^{2-}]$ = 1 mM, $c(^nBu_4N(BF_4))$ = 0.1 M) with a sweep rate of 100 mV s^{-1}. $E_{1/2}$ = +0.8 V vs. ferrocene/ferrocenium.

Supplementary Table 1. Crystallographic data and refinement parameters for **1–3** (CCDC numbers: 1431835-1431837)

	**1**	**2**	**3**
Empirical formula	$C_{48}H_{44}F_6IrO_2P_2$	$C_{20}H_{24}F_6IrN_8Zn$	$C_{48}H_{40}Cl_6IrP_2$
Formula weight	1020.97	748.08	1083.64
Temperature /K	122(1)	122(1)	122(2)
Crystal system	triclinic	tetragonal	triclinic
Space group	*P*-1	$P4_2/n$	*P*-1
a /Å	9.9800(4)	12.4516(4)	10.0669(9)
b /Å	10.9599(4)	12.4516(4)	10.1893(9)
c /Å	21.0009(8)	8.1424(3)	11.8996(11)
α /°	75.7290(10)	90	93.574(3)
β /°	78.0040(10)	90	99.116(3)
γ /°	71.3920(10)	90	115.451(3)
Volume /$Å^3$	2088.84(14)	1262.42(7)	1076.51(17)
Z	2	2	1
ρ_{calc} g/cm^3	1.623	1.9678	1.672
μ /mm^{-1}	3.339	6.285	3.582
F(000)	1018.0	720.5	537.0
Crystal size /mm^3	0.369 × 0.171 × 0.165	0.3 × 0.1 × 0.1	0.35 × 0.28 × 0.23
Radiation	Mo Kα (λ = 0.71073)	Mo Kα (λ = 0.71073)	Mo Kα (λ = 0.71073)
2θ range for data collection /°	4.35 to 66.492	4.62 to 66.16	4.476 to 61.012
Index ranges	$-15 \le h \le 15$, $-16 \le k \le 16$, $-32 \le l \le 32$	$-19 \le h \le 19$, $-18 \le k \le 19$, $-12 \le l \le 12$	$-14 \le h \le 14$, $-14 \le k \le 14$, $-16 \le l \le 16$
Reflections collected	140898	22599	111623
Independent reflections	16060 [R_{int} = 0.0482, R_{sigma} = 0.0251]	2406 [R_{int} = 0.0357, R_{sigma} = 0.0179]	6576 [R_{int} = 0.0444, R_{sigma} = 0.0144]
Data/restraints/parameters	16060/0/548	2406/0/82	6576/0/259
Goodness-of-fit on F^2	1.063	1.037	1.056
Final *R* indexes [$I \ge 2\sigma(I)$]	R_1 = 0.0187, wR_2 = 0.0409	R_1 = 0.0134, wR_2 = 0.0298	R_1 = 0.0127, wR_2 = 0.0330
Final *R* indexes [all data]	R_1 = 0.0230, wR_2 = 0.0419	R_1 = 0.0253, wR_2 = 0.0350	R_1 = 0.0128, wR_2 = 0.0331
Largest diff. peak/hole / e $Å^{-3}$	1.88/-0.53	1.16/-0.72	0.76/-0.79

Supplementary Table 2. CASSCF results.

CAS(5,3) - t_{2g}^{5} (3 doublet CSFs)

	**1**[a]	**2**[b]	**3**[c]
g_x	2.122	2.638	1.805
g_y	2.144	2.638	1.867
g_z	1.408	1.051	2.018
g_{iso}	1.891	2.109	1.897
1st exc. state (Kramers doublet)	6304 cm^{-1}	6209 cm^{-1}	5978 cm^{-1}
2nd exc. state (Kramers doublet)	7025 cm^{-1}	7341 cm^{-1}	6074 cm^{-1}

CAS(5,5) - $t_{2g}^{5}e_{g}^{0}$, $t_{2g}^{4}e_{g}^{1}$, $t_{2g}^{3}e_{g}^{2}$ (12 doublet CSFs, 6 quartet CSFs, 1 sextet CSF)

	**1**[a]	**2**[b]	**3**[c]
g_x	2.183	2.238	1.885
g_y	2.201	2.238	1.931
g_z	1.592	1.295	2.069
g_{iso}	1.992	1.924	1.962
1st exc. state (Kramers doublet)	8097 cm^{-1}	7906 cm^{-1}	7375 cm^{-1}
2nd exc. state (Kramers doublet)	8880 cm^{-1}	9223 cm^{-1}	7489 cm^{-1}

[a] $[IrF_6]^{2-}$•$2H_2O$ fragment
[b] $F[Zn(viz)_4][IrF_6][Zn(viz)_4]F$-fragment
[c] $[IrCl_6]^{2-}$ fragment
